# Theoretical Survey of the Intrinsic Reactivity of Functionalized (CH_2_=C(R)XH) Enols, Enethiols and Eneselenols: Potential Interstellar Species †

**DOI:** 10.3390/molecules31061040

**Published:** 2026-03-20

**Authors:** Al Mokhtar Lamsabhi, Otilia Mó, Jean-Claude Guillemin, Manuel Yáñez

**Affiliations:** 1 Departamento de Química, Módulo 13, Facultad de Ciencias, Associated Unit with the IQM-CSIC, Universidad Autónoma de Madrid, 28049 Madrid, Spain; mokhtar.lamsabhi@uam.es (A.M.L.); otilia.mo@uam.es (O.M.); 2 Institute for Advanced Research in Chemical Sciences (IAdChem), Universidad Autónoma de Madrid, 28049 Madrid, Spain; 3 Univ Rennes, Ecole Nationale Supérieure de Chimie de Rennes, CNRS, ISCR-UMR 6226, F-35000 Rennes, France

**Keywords:** enols, enethiols, eneselenols, G4 calculations, intrinsic reactivity, astrochemistry

## Abstract

The conformational properties and intrinsic reactivity of unsaturated CH_2_=C(R)XH systems (R = –H, –CH=CH_2_, –C≡CH, –C≡N, –Cl, –phenyl, –cyclopentadienyl, –pyrrole; X = O, S, Se)—namely enols, enethiols, and eneselenols—have been investigated using G4 and CCSD(T) calculations. All compounds exhibit antiperiplanar (*ap*) and anticlinal (*ac*)-conformers that are nearly isoenergetic, as their relative stabilities are governed by subtle noncovalent interactions, which are analyzed in detail. Both conformers are therefore expected to coexist in the gas phase, and because the rotational barriers are very low, their interconversion is effectively barrierless under typical conditions. In contrast, the corresponding protonated species display significantly higher barriers, approximately three to five times larger. The keto–enol tautomerization involves activation barriers exceeding 180 kJ·mol^−1^, confirming that, as in other keto–enol rearrangements, the process is not monomolecular. Protonation generally occurs at the methylene carbon, with the exceptions of the –C≡CH and –C≡N derivatives. Strong linear correlations are found among the proton affinities of the three families studied, which follow the trend: enols > enethiols > eneselenols. All systems behave as strong carbon bases; some are predicted to be 20–21 orders of magnitude more basic than ketene and 3–5 orders of magnitude more basic than vinylimine in terms of equilibrium constants. Deprotonation preferentially occurs at the X–H group in nearly all cases. The only exception is the cyclopentadienyl-substituted enol, for which deprotonation of the cyclopentadienyl moiety is favored due to enhanced aromatic stabilization of the resulting anion. Overall, acidity increases along the series O < S < Se.

## 1. Introduction

**Scheme 1:**
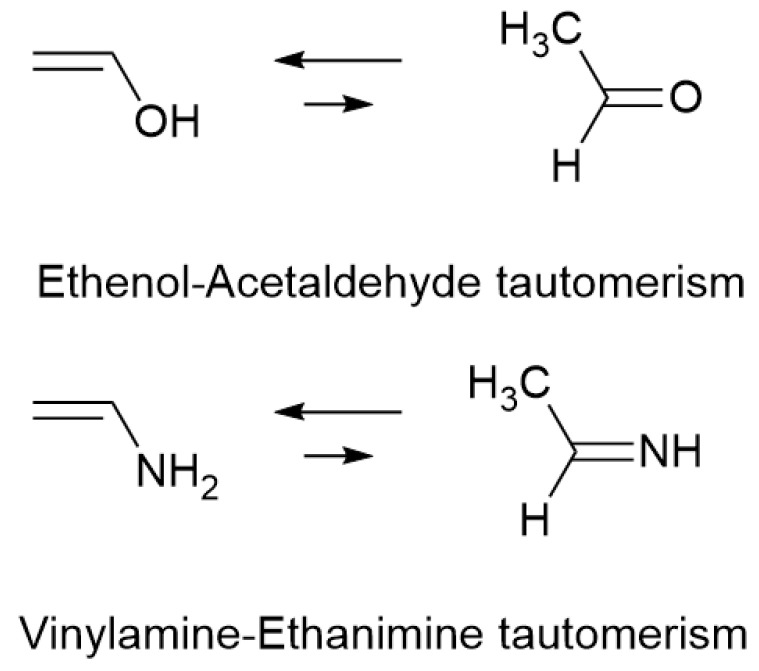
Ethenol-Acetaldehyde and Vinylamine-Ethanimine tautomerisms.

Since the discovery in 1937 of the first molecule in the interstellar medium (ISM) [1,2,3], approximately 340 molecules have been detected, with more than 20 being detected each year since 2021 [4]. Most of them, with more than 3 atoms, are organic compounds and almost all the chemical functions of organic chemistry are present. Among compounds containing an unsaturated carbon–carbon double bond, alkynes are more common than alkenes. This could be related to the easier reduction of alkenes than alkynes by hydrogen radicals in the ISM [5]. Among the fascinating alkenyl compounds detected in this medium, we can mention the detection of ethenol (vinyl alcohol) in 2001 [6], the tautomer of acetaldehyde, and, in 2021, ethenamine (vinylamine) [7], the tautomer of ethanimine (Scheme 1). Even if we cannot conclude that there is efficient tautomerization in the ISM, the presence of α,β-unsaturated compounds, well known for their particular properties [8], shows, once again, the complexity of the chemistry in this medium.

**Scheme 2:**
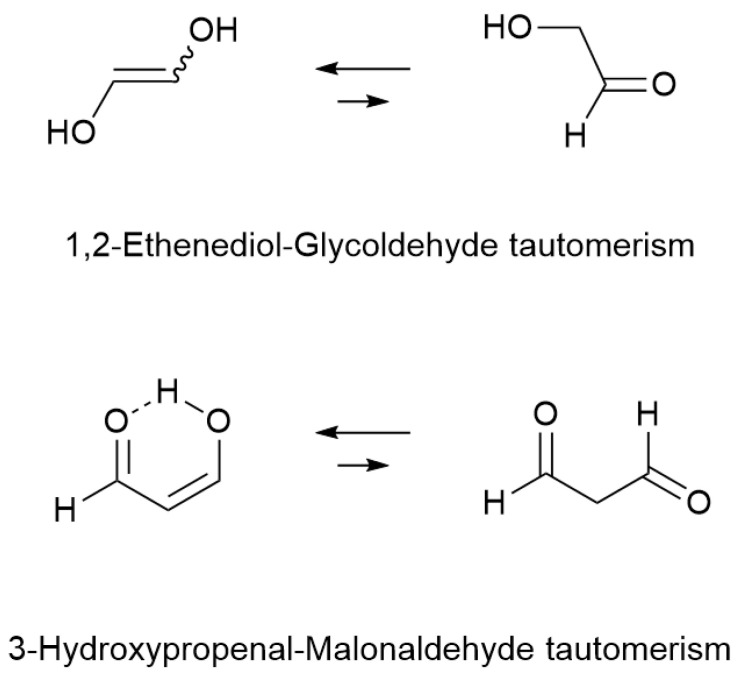
1,2-Ethenediol-Glycoldehyde and 3-Hydroxypropenal-Malonaldehyde tautomerisms.

Recently, the number of enols detected in the ISM has increased with the detection of more complex derivatives: the 1,2-ethenediol in 2022 [9], the tautomer of glycolaldehyde, a dimer of formaldehyde and a precursor of sugars, and 3-hydroxypropenal in 2022 [10], the enol of malonaldehyde, whose detection was confirmed in 2024 toward the Infrared Astronomical Satellite IRAS [11], and which is stabilized in its enol form by a hydrogen bond (Scheme 2). Of the eight compounds mentioned, only the last one (malonaldehyde) has not been detected in the interstellar medium, but the lack of laboratory recording of the microwave spectrum of this thermodynamically less stable isomer prevents its detection.

The case of sulfur derivatives is quite different, and vinylthiol (H_2_C=CH-SH) has not yet been detected in the interstellar medium. While acetaldehyde was detected there in 1973 [12], it is only very recently that the first enolizable thioaldehyde, the thioacetaldehyde (H_3_C-CH=S), has been identified in the interstellar medium [13] thanks to its spectrum recorded in the laboratory [14].

This recent identification of two functionalized enols in the ISM suggests the presence of several other enols in this medium, and the detection of the first enolizable thioaldehyde may allow us to anticipate the future detection of enethiols. However, oxygenated and sulfur-containing derivatives remain poorly studied. In such cases, reliable information on their structures, properties, and reactivity is typically obtained through accurate theoretical approaches. Numerous examples in the literature demonstrate that the structure, stability, and reactivity of small molecules of astrochemical relevance have been successfully characterized using high-level ab initio calculations [15,16,17,18,19,20,21,22]. Importantly, these methods also enable the prediction of the structures of new or yet-unobserved astrochemical compounds [23,24,25,26], including assessments of their potential existence and spectroscopic signatures [26,27,28,29,30,31,32,33,34,35,36,37,38,39,40], as well as investigations of their behavior in water or ice environments [41,42,43]. A fairly comprehensive compilation of data on a wide range of astrochemical compounds was published two years ago [44].

**Scheme 3:**
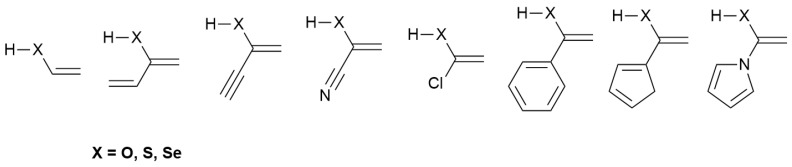
Enols, enethiols and eneselenols included in this study.

In this article, we present high-level ab initio calculations on the structure and intrinsic reactivity, namely intrinsic basicity and intrinsic acidity, of the enols and enethiols shown in Scheme 3, to which we have added, for the sake of completeness, the corresponding eneselenols. In this first paper on this kind of compound, we limited our study to the tautomers of ketones, thioketones and selenoketones, which have been much less investigated from a theoretical and experimental point of view than the corresponding tautomers of aldehydes, thioaldehydes and selenoaldehydes.

The selected set comprises a diverse range of substituents, including unsaturated alkyl groups, halogens, and both aromatic and non-aromatic cyclic moieties, as well as the unsubstituted parent compounds, enabling a systematic evaluation of substituent effects on the investigated properties. Some of these compounds—particularly the oxygen and sulfur derivatives—may be considered potential candidates for detection in the interstellar medium. This possibility constitutes one of the main motivations of the present study, as there is a complete lack of information on these enols, enethiols, and eneselenols. To the best of our knowledge, they have never been isolated, and the available literature indicates that they are unstable even at −100 °C.

## 2. Methods

A key issue in studies of this type is the accuracy of the computational methods employed. Quite often, the energy differences between conformers of a given compound fall within the range of chemical accuracy—typically 4–12 kJ·mol^−1^ —as is the case for the systems investigated here, as will be shown below. Accordingly, our survey primarily employs the well-tested high-level G4 ab initio composite method [45]. The accuracy of G4 theory has been benchmarked against the G3/05 test set [46], yielding an average absolute deviation from experimental values of 3.5 kJ·mol^−1^ [45]. In systems where the overall structure, or part of it, is governed by dispersion interactions [47], this approach may fail, since G4 relies on geometries optimized at the B3LYP level, which does not properly account for dispersion effects. In cases where dispersion is expected to be important, the G4 results are complemented by CCSD(T) calculations performed on geometries optimized with the M06-2X functional, which accounts for dispersion effects, using the flexible aug-cc-pVTZ basis set. Note that, unless otherwise stated, all proton affinities, acidities and relative enthalpies correspond to G4 electronic energies plus G4 thermal corrections at 298 K, as obtained using the Gaussian-16 program [48].

It should be noted that, for selenium derivatives, relativistic effects are undoubtedly important when evaluating properties such as spin–spin coupling constants [49], chemical shifts [50], or inner-shell ionization potentials [51]. However, their impact on optimized geometries is comparatively small, typically resulting in bond length variations of less than 0.05 Å, with average deviations on the order of 0.01–0.02 Å. Likewise, the influence of relativistic effects on the energetics of these systems is generally in the range of 4–20 kJ/mol, leading to negligible differences in relative stabilities [52].

The bonding characteristics are visualized through a topological analysis of the molecular electron density, ρ(r), within the framework of the quantum theory of atoms in molecules (QTAIM) [53]. This formalism permits us to build up the corresponding molecular graph, which contains the complete set of electron density critical points, in particular the bond critical points (BCPs) that characterize both the strength and the nature of the chemical bonds constituting the system. All these calculations have been carried out by using the AIMAll (Version 19.10.12) code [54]. The QTAIM however is not able to identify regions of low reduced density gradient (*s*) and low electron density, which are associated with noncovalent interactions. Therefore, in cases where such regions are expected to play a significant role in the properties under investigation, we will employ the NCIPLOT method [55], which is specifically designed to identify regions of low reduced density gradient (*s*) and low electron density associated with noncovalent interactions. The wavefunction obtained from G4 calculations at the B3LYP/GTBas3 level was employed for the NCIPLOT analysis. Another very useful tool for analyzing the origin of these weak interactions is the natural bond orbital (NBO) approach [56,57,58] through an inspection of the second-order perturbation terms involving occupied and empty localized molecular orbitals (LMOs).

## 3. Results and Discussion

As indicated above, some of the compounds included in this study, particularly the oxygen- and sulfur-containing derivatives, could potentially be found in the interstellar medium, where pressure and temperature conditions differ greatly from the standard conditions typically assumed in the calculations presented here. In this respect, it should be noted, however, that significant changes are not expected, since the corrections affect the different conformers in a similar manner. Indeed, for all the compounds considered, the enthalpy gaps between conformers differ only marginally from those obtained at 0 K, with an average deviation of 0.5 kJ·mol^−1^. Our calculations are not intended to simulate such environments, but rather to provide fundamental information on species for which no data currently exist regarding either their structure or their intrinsic reactivity. Nevertheless, such information may help facilitate their potential detection. In this respect we provide information on their G4 total energies, dipole moments, rotational constants and optimized geometries in the Supporting Information. Similarly, it cannot be ruled out that excited triplet states might play a relevant role in the behavior of these species under such extreme conditions. However, that analysis lies beyond the scope of the present paper, which is restricted to the singlet ground state. However, using the parent compounds as suitable test systems, we have verified that the singlet ground-state wave function is stable with respect to spin or symmetry breaking.

### 3.1. Conformational Features

**Scheme 4:**
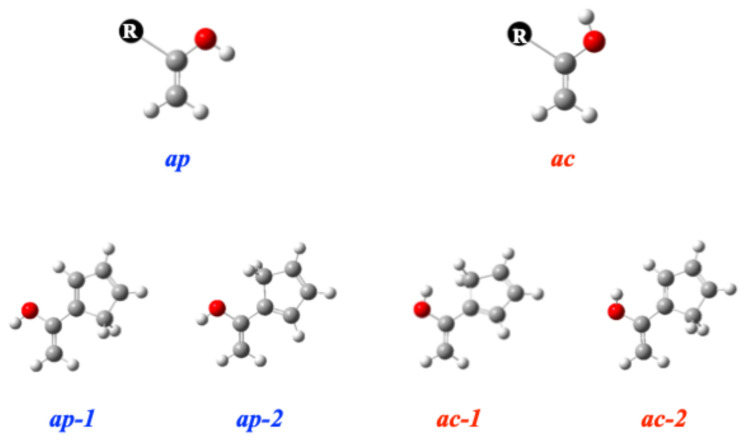
Stable conformers of CH_2_=C(R)OH (R = cyclopentadienyl) enols.

The enols, enethiols, and eneselenols considered here may adopt two conformations, antiperiplanar (*ap*) (obsolete *anti*) and anticlinal (*ac*) (obsolete *gauche*), depending on the relative orientation of the –XH hydrogen atom (X = O, S, Se) with respect to the substituent R (R = –H, –CH=CH_2_, –C≡CH, –C≡N, –Cl, –phenyl, –cyclopentadienyl, –pyrrole), as illustrated in Scheme 4.

For the CH=CH_2_ and cyclopentadienyl derivatives, a total of four conformers are possible: two *ap* and two *ac* forms. These conformers differ in the relative orientation of the substituents, as illustrated in Scheme 4 and Supplementary Figure S1.

Also importantly, the enthalpy (Gibbs free energy) differences between the *ap* and *ac* conformations are very small, the majority below 5.0 kJ·mol^−1^ and never larger than 15 kJ·mol^−1^. This suggests that both forms should coexist in the gas phase. Table 1 summarizes their relative enthalpies together with their calculated gas-phase populations at 298 K, for the three families of compounds investigated and for the various substituents considered in this study.

The stability trends are similar for enethiols and eneselenols, for which the *ac* conformer is systematically the most stable, with only two exceptions (R = –CH=CH_2_ and –cyclopentadienyl), which will be discussed in more detail later. In contrast, the stability trends for enols are less systematic. For the parent compound and for most substituents (R = –CH=CH_2,_ –phenyl, –cyclopentadienyl and –pyrrole), the *ap* conformer is favored, whereas only for R = –C≡CH, –C≡N and –Cl is the *ac* conformer found to be more stable. It should be noted, however, that in most cases the two forms are very close in energy, often so close that they can be regarded as effectively degenerate within the accuracy of the theoretical method employed. This near degeneracy can account for seemingly anomalous cases, such as the CH_2_=CHXH (X = S, Se) parent compounds and the CH_2_=CRXH (X = S, Se; R = –CH=CH_2_, –cyclopentadienyl) derivatives, in which the stability trend changes when going from enthalpies to Gibbs free energies (see Table 1). In these systems, the low-frequency vibrational modes of the two conformers differ by more than a factor of two. Because lower frequencies contribute more strongly to the Gibbs free energy through the entropic term, this difference can explain the observed stability inversion when going from enthalpies to Gibbs free energies. Nevertheless, since in these cases the *ap–ac* enthalpy differences range from 0.5 to 1.2 kJ·mol^−1^, values that are smaller than the expected accuracy of the G4 formalism, we decided to assess whether these observations simply reflect the extremely small energy separation between the two conformers, by performing reference CCSD(T)/aug-cc-pVTZ calculations using M06-2X/aug-cc-pVTZ optimized geometries for the CH_2_=CHXH (X = O, S, Se) and CH_2_=C(R)XH (X = S, Se; R = –CH=CH_2_) systems. The resulting data (Supplementary Table S1) show that the *ap–ac* enthalpy differences deviate by less than 0.5 kJ·mol^−1^ from the G4 values, and no stability inversion is observed when comparing enthalpy- and Gibbs free energy-based trends. This is likely due to small differences in the thermal vibrational corrections arising from the use of a different geometry optimization procedure.

**Figure 1:**
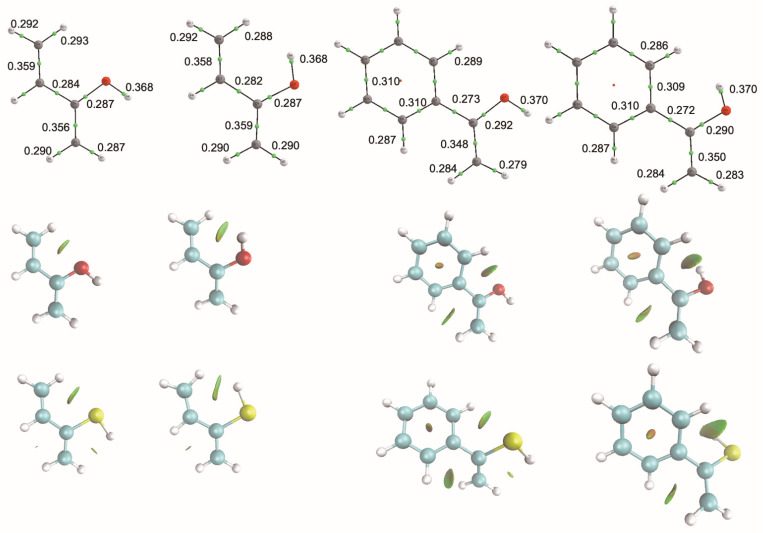
Molecular graphs and NCI plots (first and second row, respectively) for the *ap* and *ac* conformers of the CH_2_=CROH (R = –CH=CH_2_, phenyl) enols. Third row shows the NCI plots for the corresponding enethiols. Electron densities are in a.u. In the NCI plots, the green areas correspond to weak stabilizing interactions.

The greater stability of one conformer relative to the other arises from subtle differences among weak intramolecular non-covalent interactions. To discuss this point in more detail, let us compare the enol and enethiol derivatives with R = –CH=CH_2_ and R = phenyl, as suitable illustrative examples. As shown in Figure 1, and as anticipated above, the QTAIM analysis does not provide direct evidence for intramolecular NCIs, which are characterized by very small electron densities. Consequently, only conventional chemical bonds—identified by the presence of bond critical points (BCPs)—are observed in the corresponding molecular graphs. Nevertheless, some indirect indications of such NCIs can be inferred. For instance, in the *ap* conformer, the two C–H bonds of the methylene group exhibit slightly different strengths, as reflected by small differences in the electron density values at their respective BCPs.

**Figure 2:**
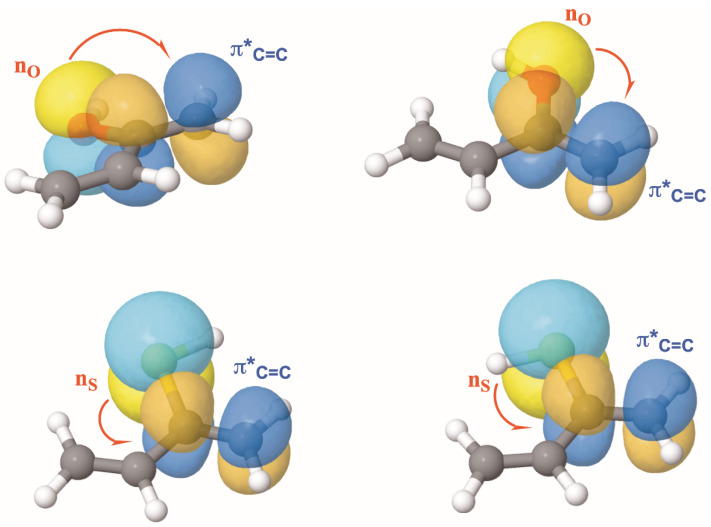
NBO localized orbitals showing the interactions between the oxygen and sulfur lone-pairs (n_O_ and n_S_, respectively) with the antibonding π*_C=C_ orbital for the ap and ac conformers of CH_2_=C(R)XH (R = –CH=CH_2_) derivatives.

This inference is in harmony with a close analysis of the possible intramolecular interactions. The NCI plots of the same systems clearly reveal the presence of intramolecular NCIs involving different functional groups. For the enols with R = –CH=CH_2_, the NCI plots show distinct interactions that differentiate the two conformers. The *ap* conformer features a stabilizing CH···OH interaction which in the *ac* conformer is replaced by a OH···HC contact. As a consequence, the ability of the oxygen lone-pair to act as an electron donor is expected to differ between the two conformations. Indeed, NBO second-order perturbation analysis (see Figure 2) shows that the dominant interaction is the donation from the oxygen lone-pair (n_O_) to the antibonding π*_CC_ orbital of the enol moiety. This interaction is ca. 24 kJ·mol^−1^ stronger in the *ap* conformer, which accordingly becomes the most stable, and exhibits a C=C bond slightly longer than the *ac* one (1.341 vs. 1.339 Å).

Turning to the corresponding enethiols, the NCI plots (see Figure 1) reveal a qualitatively similar pattern of intramolecular interactions. One might therefore expect analogous changes in the electron-donor ability of the sulfur lone pair. However, although NBO second-order perturbation analysis (Figure 2) again shows orbital interactions similar to those found in the enols, the key difference lies in their relative magnitudes. One cannot ignore that the size of the sulfur lone-pair and the length of the S–H bonds is larger and, in this case, the energy difference between the dative interaction of the sulfur lone-pair (n_S_) with the antibonding π*_CC_ orbital of the enol moiety for the *ap* and *ac* conformers nearly vanishes (ca. 3 kJ·mol^−1^), which is consistent with the G4 and CCSD(T) calculations, indicating that for CH_2_=CRSH (R = –CH=CH_2_) the two conformers are essentially degenerate.

Upon moving to the phenyl derivatives, an additional CH···HC NCI emerges between the methylene group and the aromatic ring. As shown in Figure 1, this interaction is almost identical in the *ap* and *ac* conformers. Although weak, it is expected to contribute slightly to the relative stabilities of the two conformers. For the enol, the n_O_ ⟶ π*_CC_ interaction is again stronger (ca. 36 kJ·mol^−1^) for the *ap* conformer as observed previously for the R = –CH=CH_2_ derivative. At the same time, conjugation between the C=C bond of the enol moiety and one of the π*_CC_ orbitals of the phenyl ring— associated with this new NCI attractive region— is marginally stronger (ca. 2 kJ·mol^−1^) in the *ap* conformer than in the *ac* one, consistent with the close similarity observed in the NCI plots (see Supplementary Figure S2). Accordingly, as in the R = –CH=CH_2_ case, the *ap* conformer is the most stable. The situation differs slightly, however, for the corresponding enethiols. Although the n_S_ ⟶ π*_CC_ interaction is again stronger in the *ap* than in the *ac* conformer, the energy difference is smaller (ca. 29 kJ·mol^−1^) than in the enol, and the π_CC_ ⟶ π*_CC_ interaction (Figure S2) now slightly favors the *ac* by 3 kJ·mol^−1^. Both effects together, being the dominant ones, render the *ac* conformer slightly more stable than the *ap* one.

The real presence of the different conformers depends on the size of the activation barrier associated with the internal rotation of the X–H (X = O, S, Se) group connecting them. The G4-calculated values, always referring to the most stable of the two conformers, are summarized in Supplementary Table S2. In general, all barriers are rather small, averaging ~16, ~11, and ~9 kJ·mol^−1^ for the enols, enethiols, and eneselenols, respectively. The main consequence is that the rotational barrier lies below the lowest vibrational level of the molecule because its height is smaller than the ZPE, mainly due to the large vibrational frequency associated with the X–H stretching mode, although the other vibrational modes also contribute. Overall, these results indicate that interconversion between the two conformers is effectively barrierless in practice for all the compounds analyzed.

### 3.2. Keto-Enol Tautomerism

A complete survey of the structural characteristics of the three families of compounds investigated cannot ignore that they are tautomers of the corresponding ketones formally by a H transfer from the X–H to the methylene group. The calculated keto-enol tautomerization barriers are summarized in Table 2, as well as the relative stability of the ketones with respect to the corresponding alcohols. For both magnitudes, we have used as a reference the most stable conformation of the corresponding alcohol.

It should be noted that the unimolecular tautomerization of an enol involving suprafacial-1,3-migration is “forbidden” by the Woodward-Hoffmann rules [59] because it requires very high activation energy. Ethenol can be obtained by thermolysis at very high temperatures [60], while its rearrangement to acetaldehyde is observed in solution at low temperatures, demonstrating a different mechanism. However, the values obtained by theoretical calculations reflect the energy necessary for a monomolecular rearrangement and can be used for comparison.

It can be observed that the activation barriers slightly decrease along the series OH > SH > SeH. The same qualitative trend is observed as far as the relative stability of the keto form is concerned with respect to the enol one, although for some specific substituents, –C≡CH and –C≡N, the value obtained for the eneselenol is slightly more negative than for the corresponding enethiol.

**Figure 3:**
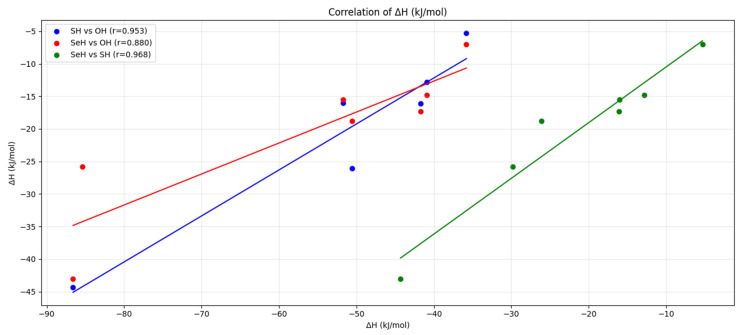
Correlation between the relative stabilities of the keto forms and the enolic forms for the three families of compounds. The correlations between the values for enethiols vs. enols and for eneselenols vs. enethiols follow the equations: ΔH (eneselenol) = 0.7066 ΔH (enethiol) + 16.087, and ΔH (eneselenol) = 0.8559 ΔH (enethiol) − 1.9254, respectively. The corresponding Pearson correlation coefficients (r) are shown in the figure.

There is a reasonable linear relationship between the activation barriers of enols and enethiols or eneselenols, though the best correlation is found between enethiols and eneselenols (See Figure S3). Curiously, such a linear correlation is not found between the relative stabilities of the keto forms when oxygen compounds are compared with sulfur or selenium ones (see Figure 3). However, the correlation is reasonably good between sulfur and selenium keto forms (see Figure 3).

The large scattering observed when correlating the relative stabilities of the keto forms of enols with those of the corresponding enethiols and eneselenols reflects significant changes in the bonding patterns of the ketone forms upon substitution of oxygen by sulfur or selenium. This effect is particularly pronounced for chlorine derivatives, which will be discussed in more detail. Here again, the second-order orbital interactions are relevant. The NBO analysis shows that in both the oxygen and the sulfur keto forms there are two significant interactions between the oxygen and sulfur lone-pairs, respectively, with the C–Cl and to a much lesser degree with the C–C antibonding orbitals, but both of them are larger for the oxygen derivatives, enhancing the stability of the keto form to a larger degree than in the enethiol. A similar effect will be found again when dealing with the deprotonated species of the chloro-derivatives of enols and enethiols (vide infra).

### 3.3. Intrinsic Reactivity

It should be emphasized that this section concerns the reactivity of isolated molecules in the gas phase. In this context, proton affinities (PAs) refer to the reaction:

Base + H^+^ → BaseH^+^ (in vacuo)

Consequently, the behavior expected in solution or for species adsorbed on ice or other condensed phases may differ substantially from the intrinsic gas-phase trends discussed here.

#### Basicity

**Figure 4:**
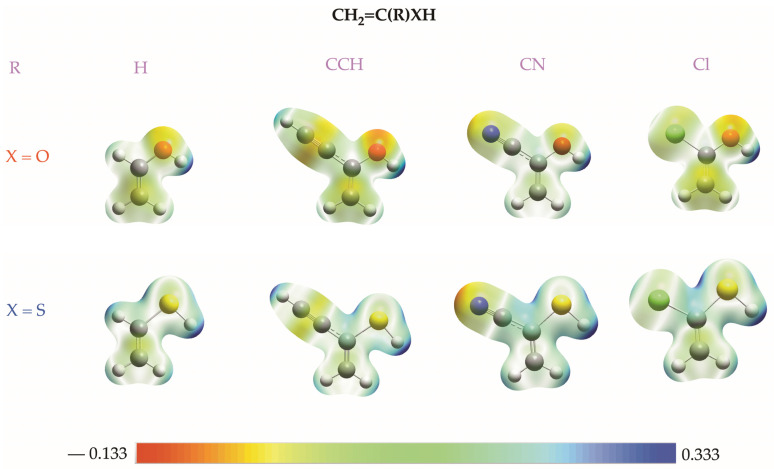
Molecular electrostatic potential for several enols and enethiols. Red and blue areas correspond to regions with negative and positive values of the potential, respectively.

Enols, enethiols, and eneselenols exhibit several potential basic sites, as evidenced by their molecular electrostatic potential (MEP) maps (Figure 4 and Figure S4).

In the parent enol, the most negative electrostatic potential is localized on the oxygen atom of the hydroxyl group, while the hydroxyl proton corresponds to the region of highest positive potential. A less intense, yet clearly negative, potential is also associated with the methylene (CH_2_) group. In the substituted derivatives, additional basic sites arise from the substituents themselves, such as the C≡C triple bond in the –CCH group, the nitrogen atom in the –CN group, and the chlorine atom in the chloro derivative. Notably, in the –CN derivative, the most negative electrostatic potential is located on the nitrogen atom of the substituent.

For enethiols, a distinct difference emerges: the most attractive electrostatic potential is not necessarily centered on the sulfur atom (see Figure 4). In several cases, more negative or comparable potentials are found at the C≡C triple bond of the –C≡CH substituent or at the methylene (CH_2_) group. Nevertheless, irrespective of the nature of X (O, S, or Se), and with the sole exceptions of the R = –C≡N and –C≡CH substituents, the CH_2_ moiety of the enol (or enethiol/eneselenol) fragment consistently emerges as the most basic site. Indeed, for the unsubstituted parent compound, protonation at the O (S, Se) atom is 94 (59, 54) kJ·mol^−1^ less exothermic than protonation at the methylene group. Likewise, protonation at the various basic sites of the substituents—again excluding R = –C≡N and R = –C≡CH—is energetically less favorable than protonation at the methylene group (see Supplementary Table S3).

**Figure 5:**
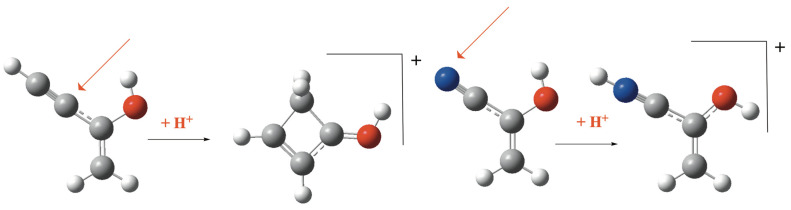
Most favorable protonation pathways for CH_2_=CROH (R = CCH. CN) compounds. Red arrows indicate the protonation sites.

As indicated above, regardless of the nature of X (O, S, or Se), the most favorable protonation process for both the R = –C≡CH and R = –C≡N derivatives occurs at the atomic sites shown in Figure 5, illustrated here using the enol systems as representative examples. Protonation of the R = –C≡CH derivatives induces cyclization, leading to a significant stabilization of the system, with the resulting cations being ca. 44, 40, and 36 kJ·mol^−1^ more stable than those formed by protonation at the methylene group in the enol, enethiol, and eneselenol, respectively. In connection with these barrierless cyclization processes, it is worth noting that similar barrierless reactions have been reported in the literature for systems with the empirical formula C_6_H_6−x_ (x = 0, 1, 2, or 3), which are of relevance to astrochemistry [61]. Another possible cyclization pathway leading to an oxirane-like ring could involve protonation at the terminal CH group. However, as shown in Figure S5, the cation formed upon this protonation is a non-cyclic structure that is 151.2 kJ·mol^−1^ less stable than the four-membered ring structure shown in Figure 5. The corresponding oxirane-like cyclic structure (Figure S5) is a further 78.2 kJ·mol^−1^ less stable. For R = –C≡N derivatives, protonation preferentially occurs at the nitrogen atom, consistent with its more negative molecular electrostatic potential. The nitrogen protonated cations of the enol, enethiol, and eneselenol are about 31, 27, and 28 kJ·mol^−1^ more stable, respectively, than the corresponding methylene-protonated species.

Discarding these two cases, the observation that protonation of the systems under scrutiny occurs at the methylene group is consistent with previous reports on related 1,1-disubstituted alkenes, such as isobutene, ketene, and vinylamine [62]. In these compounds, protonation converts the methylene group into a methyl group, thereby transforming the associated C=C double bond into a C–C single bond. As a result, *ap* and *ac* conformers—analogous to those of the corresponding neutral species—can be formed. This behavior is illustrated in Figure S6 for the phenyl, pyrrole, and chlorine derivatives as representative examples. As in the neutral systems, the enthalpy and Gibbs free energy differences between the *ap* and *ac* protonated forms are small (see Supplementary Table S3), indicating that both conformers should coexist in the gas phase at room temperature. Importantly, however, the activation barriers for interconversion between the two protonated conformers are substantially higher than those of the neutral species. Consequently, whereas interconversion between the *ap* and *ac* forms is essentially barrierless in the neutral compounds, this is not the case for the protonated systems. Both protonated conformers are therefore expected to be kinetically stable and, accordingly, detectable in the gas phase.

It is also noteworthy that protonation of the enethiols and eneselenols may lead to a reversal in the relative stability of the two conformers. For example, in enethiols and eneselenols bearing R = phenyl, pyrrole, or cyclopentadienyl substituents, the *ac* conformation is favored in the neutral species, whereas the *ap* conformation becomes more stable upon protonation. A similar *ap* preference is observed for the corresponding protonated enols; however, in this case, the *ap* arrangement is already the preferred conformation in the neutral forms. In contrast, for the chlorine-substituted derivatives, the *ac* conformation is favored in both the neutral and protonated species across all three families of compounds.

Considering that the protonated species—except for the R = –C≡CH derivative—exist in both *ap* and *ac* conformations, two proton affinities (PAs) can be reported for the compounds investigated (Table 3). These values were calculated with respect to the same conformer. In the case of R = –C≡CH, the protonated compound also has two cyclic conformations (see Supplementary Figure S7), but they are degenerate, and in this case the reported PA was calculated with respect to the most stable neutral *ac* conformer. All PA values were obtained assuming ideal gas behavior for the proton, with an enthalpy of 5RT/2 evaluated at 298 K.

Reasonably good linear correlations are observed among the proton affinities of the three families of compounds considered, particularly between the proton affinities of enethiols and eneselenols (see Figure S8). Overall, and with the sole exceptions of the R = H and C≡N derivatives, the proton affinities follow the trend enols > enethiols > eneselenols, with a slight but systematic decrease along this series. Importantly, although all these species are carbon-centered bases, they exhibit a significant intrinsic basicity. For some derivatives—particularly those with R = cyclopentadienyl and pyrrole substituents—the calculated basicities even exceed the experimental values reported for ketene (822.9 ± 3.4 kJ·mol^−1^) and vinylimine (914.0 ± 4.5 kJ·mol^−1^) [62]. Indeed, based on the changes in the corresponding Gibbs free energies, the two enols are estimated to be 20–21 orders of magnitude more basic than ketene and 3–5 orders of magnitude more basic than vinylimine in terms of the equilibrium constants.

### 3.4. Acidity

As should be expected from the characteristics of the MEPs shown in Figure 4, the less endothermic deprotonation process should be the one affecting the X–H (X = O, S, Se) group. Indeed, the alternative deprotonation of the methylene group is typically at least 140 kJ·mol^−1^ more endothermic. The loss of the proton from the substituents is similarly rather unfavorable with respect to the deprotonation of the X–H group. For instance, the substituent deprotonation of the enol derivatives where R = –C≡CH, –CH=CH_2_, phenyl, and pyrrole is 65, 195, 184, and 209 kJ·mol^−1^ less favorable than deprotonation at the X–H group, respectively.

**Figure 6:**
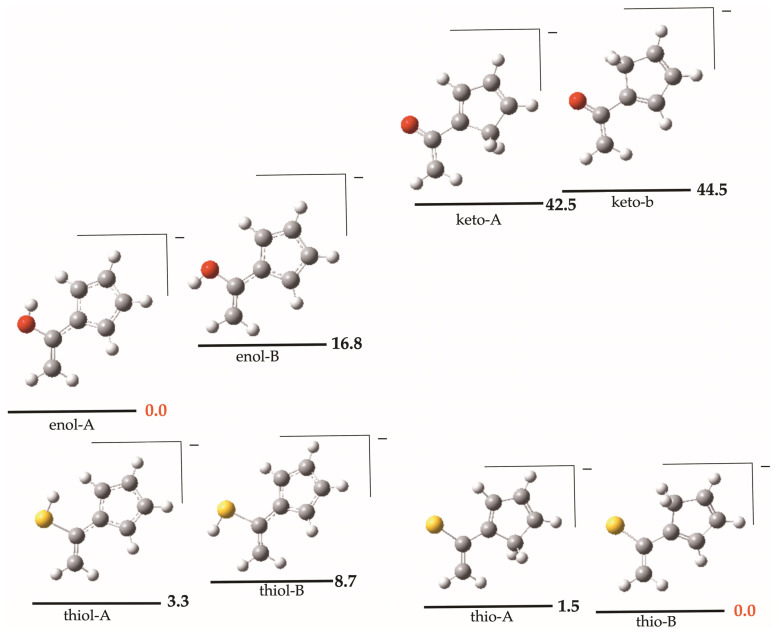
Relative stabilities (kJ·mol^−1^) of the most stable deprotonated forms of the CH_2_C(R)XH (X = O, S; R = Cyclopentadienyl).

An important exception arises exclusively for the enols: the deprotonation of the cyclopentadienyl derivative. According to our G4 calculations, loss of a proton from the saturated carbon atom of the cyclopentadienyl group—thereby promoting aromaticity in the resulting five-membered ring—is approximately 40 kJ·mol^−1^ more favorable than deprotonation of the O–H group (see Figure 6). Interestingly, a comparable stabilization is not observed for the enethiols, as shown in the lower panel of Figure 6. In this case, both deprotonation pathways are nearly equally favorable, with an enthalpy difference of only 3.3 kJ·mol^−1^, slightly favoring deprotonation at the S–H group.

**Figure 7:**
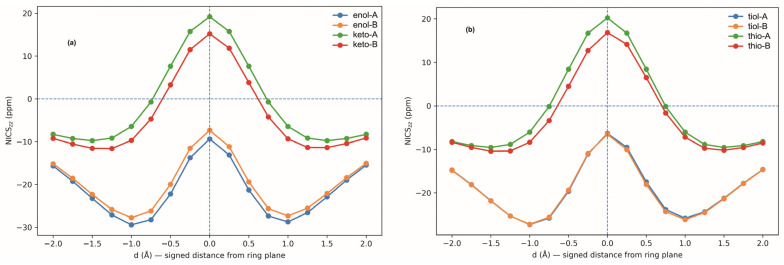
Variation in NICS as a function of the distance from the five-membered ring plane of the deprotonated forms of CH_2_=C(R)XH (R = cyclopentadienyl) (**a**) X = O, (**b**) X = S, compounds.

To understand this different behavior, we analyzed whether there is a significant difference in the aromaticity of the enol and enethiol species upon deprotonation of the cyclopentadienyl group. For this purpose, we evaluated the Nucleus-Independent Chemical Shift (NICS), defined as the negative value of the computed magnetic shielding at a selected point in space. Because the systems under investigation are not always strictly planar, NICS values were calculated both above and below the molecular plane (see Figure 7). The corresponding numerical data are summarized in Supplementary Table S4.

Inspection of the averaged NICS values indicates that both the enol and the enethiol, when deprotonated at the cyclopentadienyl substituent, exhibit clear aromatic character. The enol, however, appears to be slightly more aromatic than the enethiol. Nevertheless, this difference in aromaticity is relatively small and cannot account for the stability differences shown in Figure 6. We therefore conclude that the observed stability trends do not primarily originate from differences in aromaticity between the enol and enethiol systems.

It should be noted, however, that there is a marked difference in the charge distribution of the corresponding anions, as reflected in the changes observed in the MEPs when moving from oxygen to sulfur derivatives (see Figure S9). This difference results in significantly larger polarizabilities for the sulfur anions than for the oxygen analogues (see Table S4). These findings indicate that, beyond ring aromaticity, field-induced electronic polarization contributes to anion stabilization. Indeed, the high polarizability of the sulfur systems appears to be the dominant factor governing the relative stabilities of the enethiol anions, as evidenced by the strong correlation between their relative stabilities and their polarizabilities (see Supplementary Figure S10).

Although the anions formed by deprotonation at the X-H group cannot exhibit *ap* and *ac* conformations, two intrinsic acidity values can be evaluated depending on the conformation of the neutral. For simplicity, we report in Table 4 the acidity (Δ_acid_H^0^) calculated with respect to the most stable neutral conformer.

Limited information is available regarding the acidity of these systems. However, it is worth noting that the calculated acidities of the three parent compounds reported here are in good agreement with the G2 estimates given in Ref. [8]. For the enol parent compound, the computed Δ_acid_H° value also agrees well with the experimental value of 1490 kJ·mol^−1^ reported in Ref. [63].

**Figure 8:**
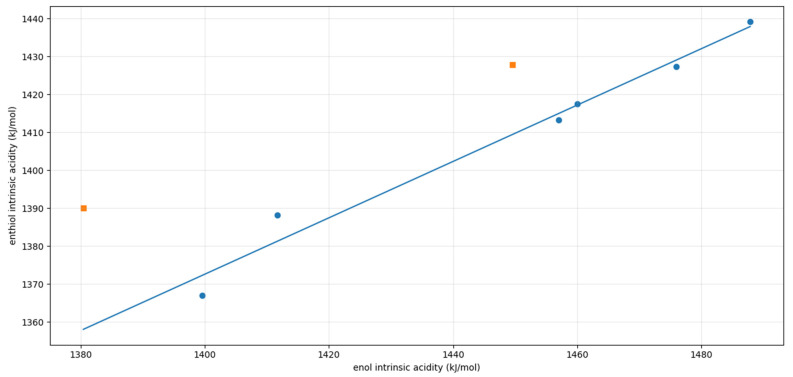
Correlation between the intrinsic acidities of enols and enethiols: IA (enethiol) = 0.7431 IA (enol) + 332.3, r = 0.988. The outlying orange points correspond to R = Cl and cyclopentadienyl derivatives, and were not included in the correlation fitting.

One unexpected feature deserves further discussion. As anticipated, the acidity increases along the series O < S < Se, and an excellent linear correlation is observed between the intrinsic acidities of enethiols and eneselenols (see Figure S11). However, as illustrated in Figure 8, the correlation is significantly poorer when comparing enols and enethiols. In this case, the acidities of the chloro- and cyclopentadienyl-derivatives deviate markedly from the overall linear trend. The deviation observed for the cyclopentadienyl derivative is not unexpected when one considers that, as discussed above, the enol behaves as a carbon acid, whereas the enethiol acts as a sulfur acid. Indeed, based on the relative stabilities shown in Figure 8, if the cyclopentadienyl enol derivative were to behave as a sulfur acid, its acidity would be 1475.4 kJ·mol^−1^. This value would fall neatly on the linear correlation shown in Figure 8.

**Figure 9:**
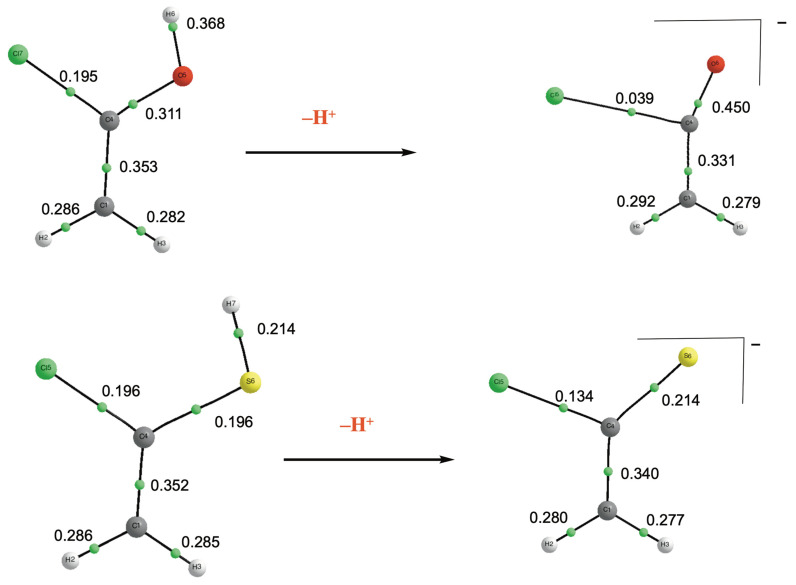
Molecular graphs of the neutral and deprotonated species of chlorine enol and chlorine enethiol derivatives. Electron densities are in a.u.

The deviation observed for the chlorine derivative must have a different origin, since both the enol and the enethiol preferentially deprotonate at the X–H group (X = O, S). However, a QTAIM analysis of the electron density reveals that the structural consequences of deprotonation differ markedly between the two species (see Figure 9). In both cases, removal of the proton from the X–H (X = O, S) group leads to a weakening of the C–Cl bond. Nevertheless, this effect is significantly more pronounced in the enol than in the enethiol, as the electron density at the corresponding BCP in the enol is about one third of the density in the enethiol. Consistently, the C–Cl bond length increases by 0.796 Å in the enol, compared to only 0.17 Å in the enethiol. This description is in harmony with the NBO analysis that shows that, in the enethiol anion, a donor–acceptor interaction between the sulfur lone-pair (n_S_) and the σ*_C–Cl_ antibonding orbital accounts for the elongation of the C–Cl, while the enolic anion is described as the result of the interaction of a H_2_C=C=O neutral moiety with a chlorine anion, reflecting a fundamentally different electronic structure.

To demonstrate that these geometric distortions are responsible for the deviation of the chlorine derivatives from the correlation, we calculated the stability of both anions while constraining the C–Cl bond length to the value found in the corresponding neutral species. The results show that the stability of the enol anion decreases by approximately 30 kJ·mol^−1^, whereas that of the enethiol anion decreases by only about 4 kJ·mol^−1^. This indicates that, in the absence of C–Cl bond distortion, the red data point corresponding to the chlorine derivatives in Figure 9 would shift horizontally to the right by 30 kJ·mol^−1^ and vertically downward by 4 kJ·mol^−1^, thereby falling in good agreement with the correlation.

## 4. Concluding Remarks

The conformational preferences of the enols, enethiols, and eneselenols investigated—CH_2_=C(R)XH (R = –H, –CH=CH_2_, –C≡CH, –C≡N, –Cl, –phenyl, –cyclopentadienyl, –pyrrole; X = O, S, Se)—are governed by subtle differences in noncovalent interactions that stabilize *ap* and *ac* conformers that are nearly isoenergetic. As a result, both conformers are expected to coexist in the gas phase. However, the rotational barriers connecting these two forms are very low, making their interconversion effectively barrierless under normal conditions.

In contrast, the corresponding protonated species exhibit significantly higher rotational barriers—approximately three to five times larger—so both protonated conformers are expected to be kinetically stable and, therefore, experimentally detectable in the gas phase. Interestingly, the relative stability ordering of the *ap* and *ac* conformers observed for the neutral species is not necessarily preserved upon protonation.

For the neutral systems, keto–enol tautomerization involves activation barriers exceeding 180 kJ·mol^−1^, confirming that, as in other keto–enol rearrangements, the process is not monomolecular. Irrespective of the nature of X (O, S, or Se), the keto forms are systematically more stable than the corresponding enol forms; however, for enethiols and eneselenols the enthalpy difference can be smaller than 10 kJ·mol^−1^.

Protonation generally occurs at the methylene carbon, with the exceptions of the –C≡CH and –C≡N derivatives. Strong linear correlations are found among the proton affinities of the three families studied, which follow the trend enols > enethiols > eneselenols, with the sole exceptions of the R = H and C≡N derivatives. All systems behave as strong carbon bases; some are predicted to be 20–21 orders of magnitude more basic than ketene and 3–5 orders of magnitude more basic than vinylimine in terms of equilibrium constants. For the R = –C≡CH derivatives, the most stable protonated structure is a cyclic species formed by proton attachment to the –C≡CH substituent.

Deprotonation preferentially occurs at the X–H group in nearly all cases. The only exception is the cyclopentadienyl-substituted enol, for which deprotonation of the cyclopentadienyl moiety is favored due to enhanced aromatic stabilization of the resulting anion. For the corresponding enethiol and eneselenol derivatives, however, deprotonation at the X–H group (X = S, Se) remains slightly more favorable than deprotonation of the cyclopentadienyl ring.

The anion formed upon deprotonation of the R = –Cl enol-derivative can be described as a complex between a neutral H_2_C=C=O moiety and Cl^−^, since deprotonation is accompanied by essentially complete C–Cl bond cleavage—a process not observed for the corresponding enethiol or eneselenol derivatives. Overall, acidity increases along the series O < S < Se.

## Figures and Tables

**Table 1:** Relative enthalpies (ΔH, kJ·mol^−1^), Gibbs free energies (ΔG, kJ·mol^−1^) and gas-phase populations of the *ap* and *ac* conformers of (CH_2_=C(R)XH) enols, enethiols and eneselenols. Reference values are highlighted in red for systems in which the *ap* conformer is the most stable, and in blue for those in which the *ac* conformer is the most stable.

Substituent		Enols			Enethiols			Eneselenols		
	Conformer	ΔH	ΔG	%	ΔH	ΔG	%	ΔH	ΔG	%
H	*ap*	0.0	0.0	79.0	0.0	0.6	44	0.0	1.1	61
	*ac*	4.4	3.3	21.0	1.1	0.0	56	0.5	0.0	39
CH=CH_2_	*ap-1*	0.0	0.0	87.4	0.4	0.0	61	0.6	0.0	58
	*ap-2*	11.1	9.9	1.6	10.1	9.9	1	10.3	9.7	1
	*ac-1*	5.5	5.2	10.7	0.0	1.4	35	0.0	1.1	37
	*ac-2*	15.8	14.4	0.3	7.3	7.3	3	7.3	7.0	4
C≡CH	*ap*	3.3	3.9	17.0	1.8	2.5	27	1.0	1.5	35
	*ac*	0.0	0.0	83.0	0.0	0.0	73	0.0	0.0	65
C≡N	*ap*	1.6	2.7	25.0	3.0	3.6	19	2.3	2.9	24
	*ac*	0.0	0.0	75.0	0.0	0.0	81	0.0	0.0	76
Cl	*ap*	4.4	9.2	2.4	1.9	1.8	33	0.9	0.8	42
	*ac*	0.0	0.0	97.6	0.0	0.0	67	0.0	0.0	58
phenyl	*ap*	0.0	0.0	86.0	2.2	2.0	31	1.8	1.6	34
	*ac*	4.0	4.6	14.0	0.0	0.0	69	0.0	0.0	66
cyclopentadienyl	*ap-1*	0.0	0.0	76.5	0.9	0.0	63.4	1.2	0.0	58.8
	*ap-2*	6.5	4.2	14.1	7.3	7.8	2.7	0.7	7.6	2.7
	*ac-1*	14.2	13.0	0.4	6.5	7.4	3.2	11.5	10.6	0.8
	*ac-2*	5.2	5.3	9.0	0.0	1.8	30.7	0.0	1.1	37.7
–pyrrole	*ap*	0.0	0.0	74.0	3.7	3.3	21	2.8	2.5	27
	*ac*	2.3	2.6	26.0	0.0	0.0	79	0.0	0.0	73

**Table 2:** G4 activation barriers for the keto–enol tautomerization process and relative enthalpies of the keto tautomers. All values (in kJ·mol^−1^) are given relative to the most stable conformer of the enol, enethiol and eneselenol compounds, respectively.

Substituent	Enols		Enethiols		Eneselenols	
	TS	Keto	TS	Keto	TS	Keto
–CH=CH_2_	228.5	−41.7	219.7	−16.1	210.4	−17.3
–C≡CH	235.3	−40.9	222.8	−12.8	211.4	−14.8
–C≡N	249.8	−35.8	235.4	−5.3	223.8	−7.0
–Cl	221.7	−85.4	215.1	−29.8	205.9	−25.8
–phenyl	214.8	−51.7	206.7	−16.0	196.1	−15.5
–c-pentadienyl	216.1	−50.6	204.9	−26.1	192.6	−18.8
–pyrrole	189.0	−86.6	185.1	−44.3	175.9	−43.0

**Table 3:** G4 proton affinities (kJ·mol^−1^) of the enols, enethiols and eneselenols included in this study ^a^.

Substituent	Conformer	Enols	Enethiols	Eneselenols
H	*ap*	814.7	817.9	815.6
	*ac*	817.1	818.8	815.9
CH=CH_2_	*ap-1*	871.6	859.3	854.3
	*ap-2*	881.9	875.0	870.7
	*ac-1*	869.2	856.9	852.1
	*ac-2*	886.6	873.9	869.2
C≡CH	*cyclic*	880.4	877.5	873.4
C≡N	*ap*	773.8	783.1	789.5
	*ac*	772.2	780.1	787.2
Cl	*ap*	826.9	822.7	820.9
	*ac*	830.0	823.5	822.2
phenyl	*ap*	909.1	892.8	886.8
	*ac*	908.0	887.2	881.7
cyclopentadienyl	*ap-1*	930.5	917.2	911.9
	*ap-2*	941.6	930.2	925.3
	*ac-1*	942.6	926.0	926.2
	*ac-2*	927.0	912.6	908.0
pyrrole	*ap*	932.7	918.8	915.5
	*ac*	925.4	909.2	907.2

^a^ All systems protonate at the methylene group, with the exception of those with R = C≡CH and C≡N (see text).

**Table 4:** G4 intrinsic acidities (Δ_acid_H^0^, kJ·mol^−1^) of the enols, enethiols and eneselenols included in this study.

Substituent	Enols	Enethiols	Eneselenols
H	1494.1	1445.4	1414.5
CH=CH_2_	1482.2	1433.6	1405.6
C≡CH	1463.2	1419.5	1392.2
C≡N	1405.7	1373.2	1348.7
Cl	1386.6	1396.2	1373.9
phenyl	1466.2	1423.7	1396.7
cyclopentadienyl	1455.8	1434.1	1405.1
pyrrole	1417.9	1394.3	1370.7

## Data Availability

The authors confirm that the data supporting the findings of this study are available within the article and its Supplementary Materials.

## Supplementary Materials

The following supporting information can be downloaded at: https://www.mdpi.com/article/10.3390/molecules31061040/s1; Stable conformers of CH_2_=C(R)OH (R = CH=CH_2_, cyclopentadienyl), CCSD(T)/aug-cc-pVTZ relative enthalpies, and Gibbs free-energies for CH_2_=CHXH (X = S, Se) and CH2=CRXH (X = S, Se; R = –CH=CH_2_) systems. NBO localized orbitals showing the interactions between the oxygen and sulfur lone-pairs (n_O_ and n_S_, respectively) with the antibonding π*_CC_ orbital and between bonding of the π_C=C_ to the antibonding π*_C=C_ orbital for the *ap* and *ac* conformers of CH_2_=C(R)XH (X = O, S; R = –phenyl) derivatives. G4 activation barriers for the *ap-ac* tautomerization. Linear correlations between the keto–enol activation barriers for the enols, enethiols and eneselenols. The molecular electrostatic potential for several enols and enethiols. Relative enthalpies, Gibbs free energies and gas-phase populations of the *ap* and *ac* conformers of the protonated (CH_2_=C(R)XH) enols, enethiols and eneselenols Structures associated with the protonation at the terminal CH group of the CH_2_=C(R)OH (R = –C≡CH) derivative. Structures of the protonated species of the enethiols and eneselenols included in this study, Conformers obtained upon protonation of CH_2_=C(R)XH (R = –CCH, X = O, S). Linear correlations between the G4 PAs of the enols, enethiols and eneselenols. The molecular electrostatic potential for the different deprotonated species of the CH_2_C(R)XH enols and enethiols (R = cyclopentadienyl). NICS and polarizabilities α_iso_ for the anions included in Figure 7. Linear correlation between the polarizability (α_iso_) and the relative enthalpies (ΔH) of the deprotonated species of CH_2_C(R)SH (R = cyclopentadienyl). Linear correlation between the intrinsic acidities of enethiols and eneselenols, G4-total energies, dipole moments, rotational constants and optimized geometries.
