# Supplementary material for: Theoretical Survey of the Intrinsic Reactivity of Functionalized (CH2=C(R)XH) Enols, Enethiols and Eneselenols: Potential Interstellar Species
Source: Molecules. 2026 Mar 20;31(6):1040. doi: 10.3390/molecules31061040 (PMC13029071; doi:10.3390/molecules31061040)
Supplement: Supplementary file 1 [file molecules-31-01040-s001.zip › molecules-4189411-supplementary.pdf]

Theoretical Survey of the Intrinsic Reactivity of Functionalized (CH_2=C(R)XH) Enols, Enethiols and Eneselenols: Potential Interstellar Species

Supporting Information

CONTENTS

Figure S1. Stable conformers of CH_2=C(R)OH (R = cyclopentadienyl)

Table S1. CCSD(T)/aug-cc-pVTZ relative enthalpies and Gibbs free-energies for CH_2=CHXH (X = S, Se) and CH_2=CRXH (X = S, Se; R = –CH=CH_2) systems.

Figure S2. NBO localized orbitals showing the interactions between the oxygen or sulfur lone-pairs (n_O and n_S, respectively) with the antibonding π^*_{CC} orbital and between the bonding $\pi_{C=C}$ to the antibonding $\pi^*_{C=C}$ orbital for the *ap* and *ac* conformers of CH_2=C(R)XH (X = O, S; R = –phenyl) derivatives.

Table S2. G4 activation barriers for the *ap-ac* tautomerization**.**

Figure S3. Linear correlations between the keto-enol activation barriers for the enols, enethiols and eneselenols included in this study.

Figure S4. Molecular electrostatic potential for several enols and enethiols.

Table S3. Relative enthalpies, Gibbs free energies and gas-phase populations of the *ap* and *ac* conformers of the protonated (CH_2=C(R)XH) enols, enethiols and eneselenols.

Figure S5. Structures associated to the protonation at the terminal CH group of the CH_2=C(R)OH (R = –C≡CH) derivative.

Figure S6. Structures of the protonated species of the enethiols and eneselenols included in this study.

Figure S7. Conformers obtained upon protonation of CH_2=C(R)XH (R = –CCH, X = O, S).

Figure S8. Linear correlations between the G4 PAs of the enols, enethiols and eneselenols included in this study.

Figure S9. Molecular electrostatic potential for the different deprotonated species of the CH_2C(R)XH enols and enethiols (R = cyclopentadienyl).

Table S4. NICS and polarizabilities α_{iso} for the anions included in Figure 8

Figure S10. Linear correlation between the polarizability (α_{iso}) and the relative enthalpies (ΔH) of the deprotonated species of CH_2C(R)SH (R = cyclopentadienyl).

Figure S11. Linear correlation between the intrinsic acidities of enethiols and eneselenols

G4-total energies, dipole moments, rotational constants and optimized geometries

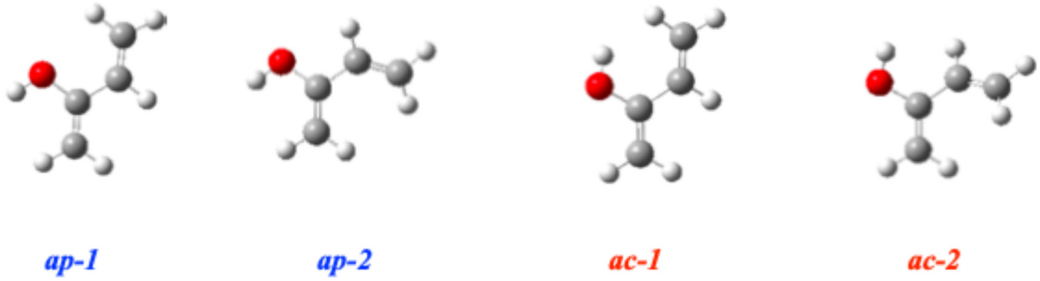

Figure S1. Stable conformers of CH_2=C(R)OH (R = CH=CH_2). Analogous conformers are also found to be stable for the corresponding enethiol and eneselenol derivatives.

Table S1. CCSD(T)/aug-cc-pVTZ relative enthalpies and Gibbs free-energies for CH_2=CHXH (X =O, S, Se) and CH_2=C(R)XH (X = S, Se; R = –CH=CH_2) systems. Reference values are highlighted in red for systems in which the *ap* conformer is the most stable, and in blue for those in which the *ac* conformer is the most stable.

system	ΔH	ΔG
OH_H_*ap*	**0.0**	**0.0**
OH_H_*ac*	5.0	3.9
SH_H_*ap*	**0.0**	**0.0**
SH_H_*ac*	1.5	0.7
SH_C_2H_3_*ap*	0.6	0.2
SH_C_2H_3_*ac*	**0.0**	**0.0**
SeH_H_*ap*	**0.0**	**0.0**
SeH_H_*ac*	0.7	1.5
SeH_C_2H_3_*ap*	1.9	1.4
SeH_C_2H_3_*ac*	**0.0**	**0.0**

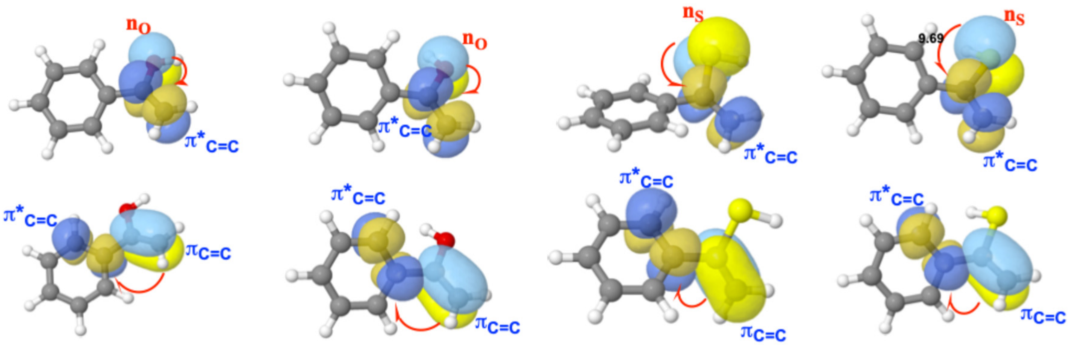

Figure S2: NBO localized orbitals showing the interactions between the oxygen or sulfur lone-pairs (n_O and n_S, respectively) with the antibonding π^*_{CC} orbital and between the bonding $\pi_{C=C}$ to the antibonding $\pi^*_{C=C}$ orbital for the *ap* and *ac* conformers of CH_2=C(R)XH (X = O, S; R = –phenyl) derivatives.

Table S2. G4 activation barriers for the *ap-ac* interconversion relative to the most stable neutral and protonated species. All values in kJ·mol^{-1}.

Substituent	**enols**		**enethiols**		**eneselenols**	
	neutral[a]	protonated	neutral[a]	protonated	neutral[a]	protonated
–H	19.7	100.4	10.6	162.7	10.2	157.0
–CH=CH_2	20.4	79.6	7.7	100.2	5.9	93.3
–C≡CH	18.3	–[a]	11.2	–[a]	8.2	–[a]
–C≡N	16.8	–[b]	12.0	–[b]	7.8	–[b]
–Cl	16.3	81.8	9.8	111.9	7.6	105.4
–phenyl	18.1	66.5	16.4	76.9	10.2	69.7
–c-pentadienyl	21.7	59.2	12.3	59.9	10.4	52.8
–pyrrole	15.0	48.1	10.0	50.2	14.0	44.3

[a] This compound forms a cycle upon protonation.
[b] This compound protonates at the N atom rather than at the methylene group.

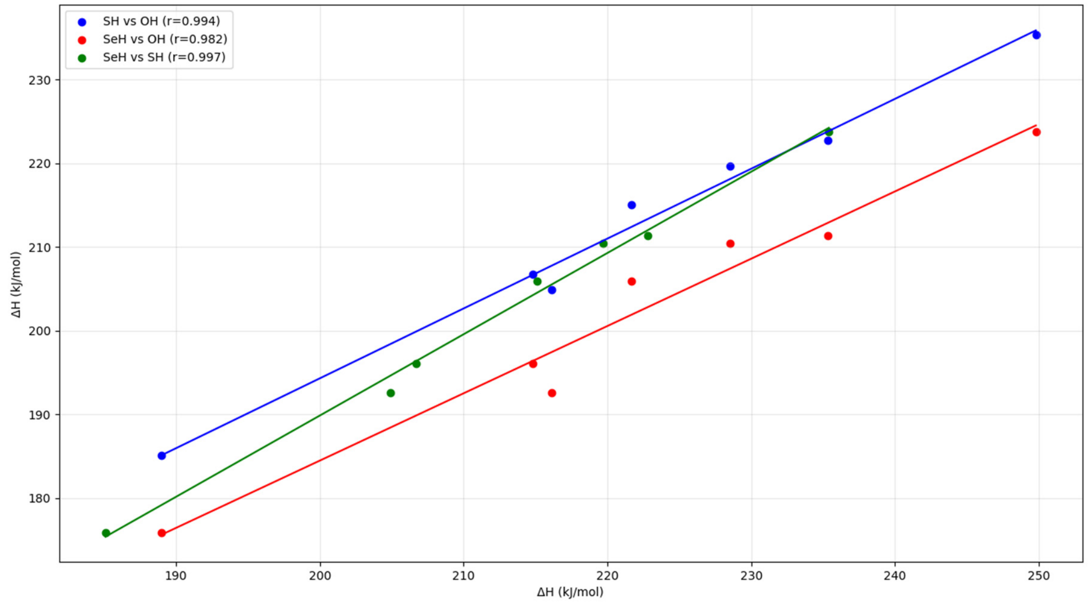

Figure S3. Linear correlations between the keto-enol activation barriers (kJ·mol^{-1}) for the enols, enethiols and eneselenols included in this study. The correlations shown obey the equations: ΔH (enethiol) = 0.8351 ΔH (enol) + 27.283; ΔH (eneselenol) = 0.8043 ΔH (enol) + 23.604; ΔH (eneselenol) = 0.9714 ΔH (enethiol) – 4.4286. The corresponding Pearson correlation coefficients (r) are shown in the figure.

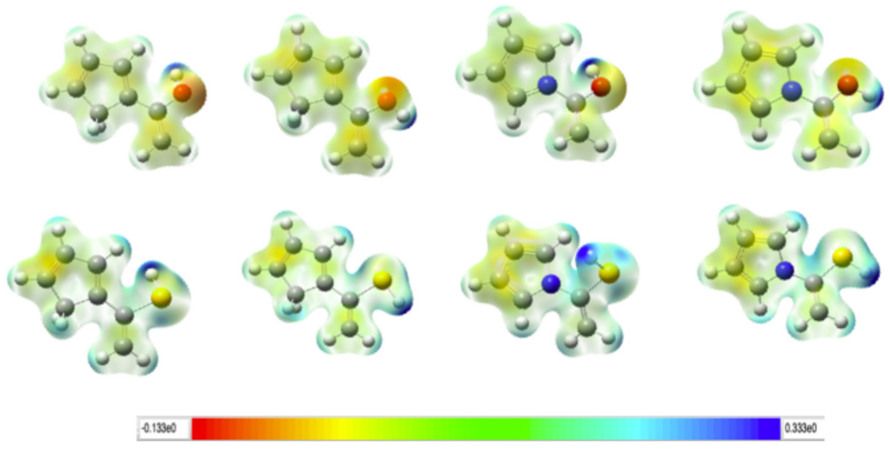

Figure S4. Molecular electrostatic potential for several enols and enethiols. Red and blue areas correspond to the nucleophilic and electrophilic regions, respectively.

Table S3. Relative enthalpies, Gibbs free energies and gas-phase populations of the *ap* and *ac* conformers of the protonated (CH_2=C(R)XH) enols, enethiols and eneselenols. Reference values are highlighted in red for systems in which the *ap* conformer is the most stable, and in blue for those in which the *ac* conformer is the most stable.

Substituent		Enols			Enethiols			Eneselenols		
	Tautomer	ΔH	ΔG	%	ΔH	ΔG	%	ΔH	ΔG	%
H	*ap*	**0.0**	**0.0**	70	**0.0**	**0.0**	62	**0.0**	**0.0**	62
	ac	2.1	2.1	30	0.2	0.3	38	0.2	0.3	38
–CH=CH_2	*ap-1*	**0.0**	1.2	25	7.7	4.1	10	8.2	6.4	4
	ap-2	0.8	0.5	33	1.8	**0.0**	50	1.6	**0.0**	53
	ac-1	8.0	6.5	3	9.7	9.0	1	9.8	9.2	1
	ac-2	0.7	**0.0**	39	**0.0**	0.6	39	**0.0**	0.6	42
–C≡N[a]	*ap*	**0.0**	**0.0**	80	—	—	—	—	—	—
	ac	10.8	3.4	20	—	—	—	—	—	—
–Cl	*ap*	7.5	7.3	5	2.8	2.1	30	2.2	1.5	35
	ac	**0.0**	**0.0**	95	**0.0**	**0.0**	70	**0.0**	**0.0**	65
–phenyl	*ap*	**0.0**	**0.0**	66	**0.0**	**0.0**	95	**0.0**	**0.0**	97
	ac	5.1	1.6	34	3.4	7.4	5	3.3	8.7	3
–cyclopentadienyl	*ap-1*	**0.0**	**0.0**	77.2	**0.0**	**0.0**	67.9	**0.0**	**0.0**	64.9
	ap-2	4.6	4.3	13.6	6.6	5.5	7.3	6.9	5.6	6.8
	ac-1	13.3	11.1	0.9	10.4	8.8	1.9	9.7	7.9	2.7
	ac-2	6.7	5.5	8.3	3.4	2.7	22.8	2.9	2.3	25.6
–pyrrole	*ap*	**0.0**	**0.0**	98	**0.0**	**0.0**	70.9	**0.0**	**0.0**	80.5
	ac	9.6	9.7	2	6.0	2.2	29.1	5.5	3.5	19.5

[a] For enethiols and eneselenols, protonation of the R = CN derivatives at the nitrogen atom yields a cation in which the X–H group is nearly perpendicular to the molecular plane; consequently, this structure cannot be classified as either *ap* or *ac*.

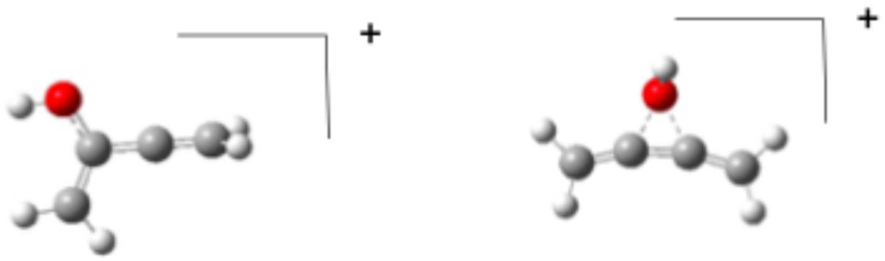

Figure S5. Structures associated to the protonation at the terminal CH group of the CH_2=C(R)OH (R = –C≡CH) derivative.

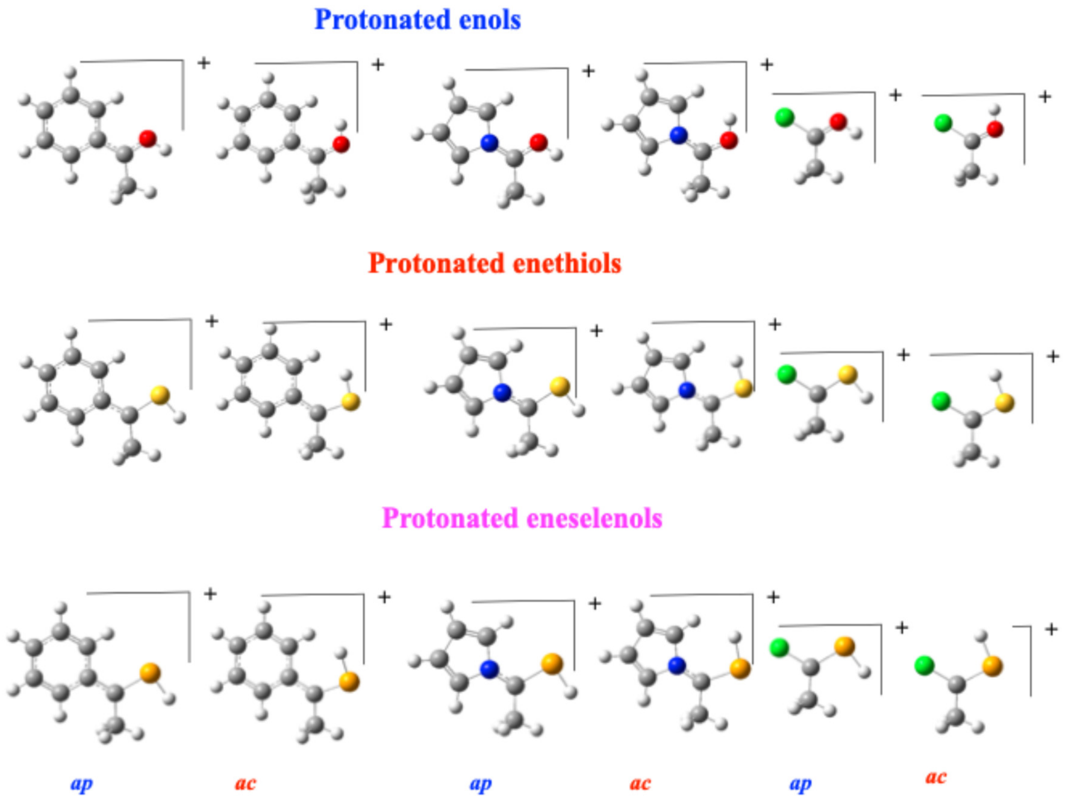

Figure S6. Structures of the two most stable protonated conformers (*ap* and *ac*) of CH_2=C(R)XH compounds (X = O, S, Se; R = phenyl, pyrrole, chlorine).

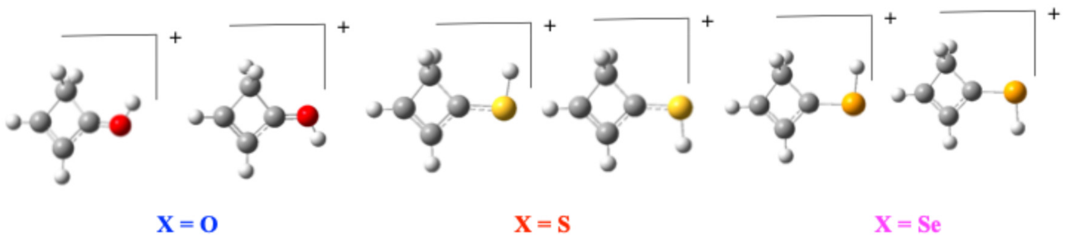

Figure S7. Conformers obtained upon protonation of CH_2=C(R)XH (R = –CCH, X = O, S)

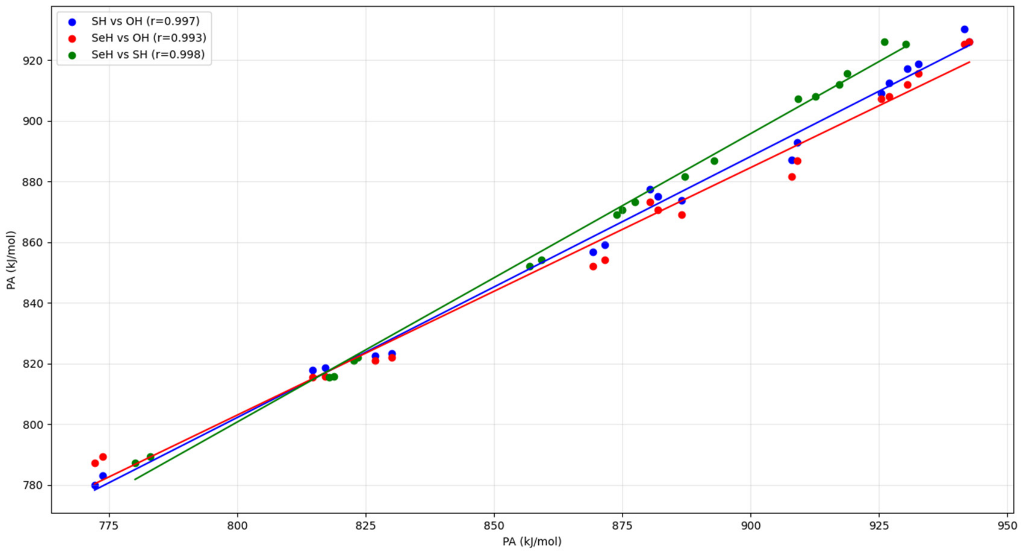

Figure S8. Linear correlations between the G4 PAs of the enols, enethiols and eneselenols included in this study. The correlations shown obey the equations: PA (enethiol) = 0.8611 PA (enol) + 113.65, r = 0.996; PA (eneselenol) = 0.8166 PA (enol) + 150.05, r = 0.993; PA (eneselenol) = 0.9501 PA (enethiol) + 40.825, r = 0.998.

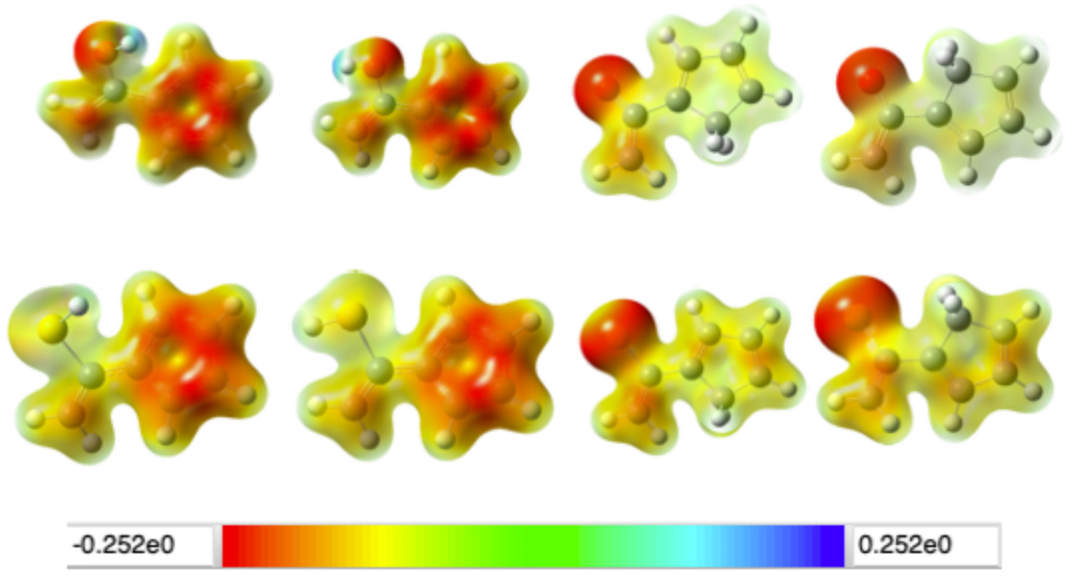

Figure S9. Molecular electrostatic potential for the different deprotonated species of the $CH_2C(R)XH$ enols and enethiols (R = cyclopentadienyl). Red and blue areas correspond to the nucleophilic and electrophilic regions, respectively.

Table S4. NICS and polarizabilities α_{iso} for the anions included in Figure 8

	**NICSzz(+1) (ppm)**	**NICSzz(-1) (ppm)**	**NICS$_{eff}$(1) (ppm)**	α_{iso}_(a.u.)
**Enol-A**	-28.7372	-29.4359	-29.0865	115.538
**Enol-B**	-27.3431	-27.7721	-27.5576	118.598
**Keto-A**	-9.3332	-9.70542	-9.51931	112.251
**Keto-B**	-6.4515	-6.45093	-6.45120	113.596
**Thiol-A**	-26.1573	-27.2289	-26.6931	135.814
**Thiol-B**	-25.8452	-27.2956	-26.5703	138.077
**Thio-A**	-7.1977	-8.3723	-7.7850	133.769
**Thio-B**	-6.0375	-6.0383	-6.0379	133.312

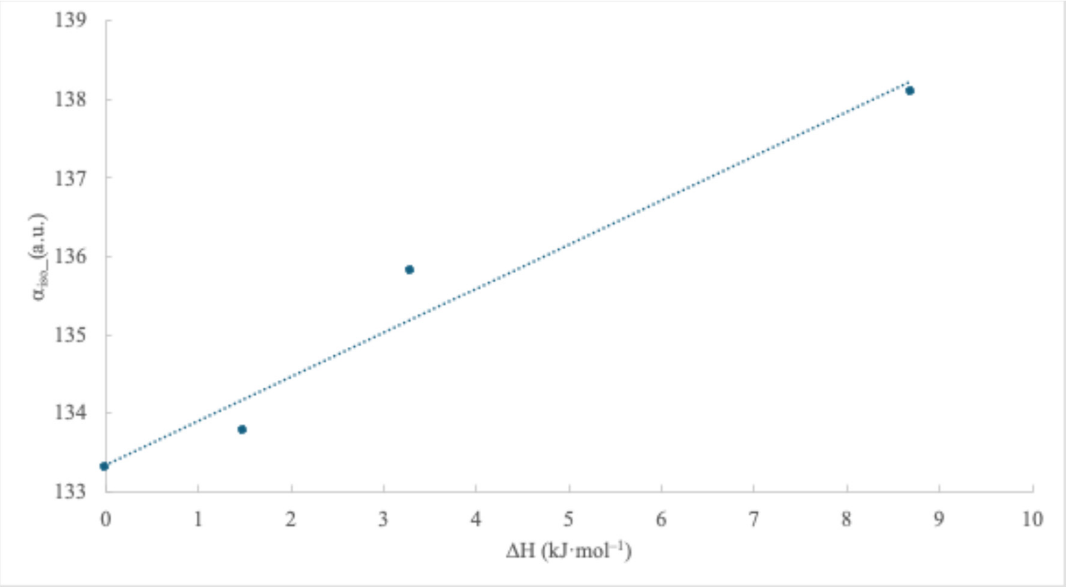

Figure S10. Linear correlation between the polarizability (α_{iso}) and the relative enthalpies (ΔH) of the deprotonated species of $CH_2C(R)SH$ (R = cyclopentadienyl). The correlation obeys the equation: $\alpha_{iso} = 0.5623\ \Delta H + 133.35$, $R^2 = 0.959$

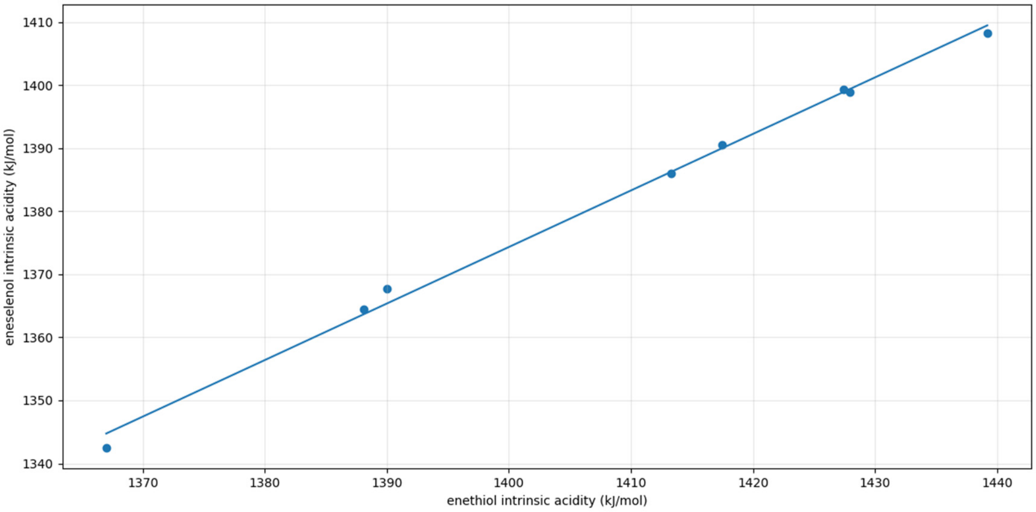

Figure S11. Linear correlation between the intrinsic acidities of enethiols and eneselenols: IA (eneselenol) = 0.8967 IA (enethiol) + 118.92, r = 0.998

G4-total energies (a.u.), dipole moments, rotational constants and optimized geometries (Å) obtained at the B3LYP/ GTBas3

Enols

R = C_2H_3 Conformer *ac*

G4 energy: -231.0744720000; Dipole moment = 1.8382 Debye; Rotational constants (MHz), A=9828.49 B=4246.72 C=2979.33

atom	X	Y	Z
C	1.684854	-0.714059	-0.042290
H	1.670373	-1.794630	-0.081926
H	2.640904	-0.208394	-0.079990
C	0.538560	-0.024799	0.017016
O	0.571205	1.341467	-0.003330
H	-0.255508	1.675205	0.360303
C	-0.767404	-0.682605	0.067898
H	-0.728433	-1.756399	0.227783
C	-1.950716	-0.078324	-0.064291
H	-2.049493	0.984328	-0.267638
H	-2.879245	-0.633127	-0.001894

R = C_2H_3 Conformer *ap*

G4 energy: -231.0765680000; Dipole moment = 0.8030 Debye; Rotational constants (MHz), A=9949.95 B=4276.11 C=2990.78

atom	X	Y	Z
C	-0.602564	1.712461	0.000000
H	-1.681626	1.782328	0.000000
H	-0.042664	2.641514	0.000000
C	0.000000	0.514908	0.000000
O	1.354221	0.346055	0.000000
H	1.774648	1.212246	0.000000
C	-0.741343	-0.744223	0.000000
H	-1.821799	-0.634292	0.000000
C	-0.183641	-1.955723	0.000000
H	0.892654	-2.080398	0.000000
H	-0.789690	-2.854371	0.000000

R = CCH Conformer *ac*

G4 energy: -229.8412580000; Dipole moment = 1.6800 Debye; Rotational constants (MHz), A=10545.00 B=4152.79 C=2979.44

atom	X	Y	Z
C	-0.992854	-1.367072	0.000000
H	-2.021621	-1.038507	0.000000
H	-0.777983	-2.428029	0.000000
C	0.000000	-0.472566	0.000000
O	1.308284	-0.881013	0.000000
H	1.872340	-0.101400	0.000000
C	-0.212571	0.938178	0.000000
C	-0.313052	2.138405	0.000000
H	-0.428145	3.194366	0.000000

R = CCH Conformer *ap*

G4 energy: -229.8400090000; Dipole moment = 1.2879 Debye; Rotational constants (MHz), A=10641.98 B=4128.83 C=2974.71

atom	X	Y	Z
C	-1.063660	-1.259836	0.000000
H	-2.064009	-0.852262	0.000000
H	-0.953315	-2.339087	-0.000000
C	0.000000	-0.446422	-0.000000
O	1.302275	-0.859396	-0.000000
H	1.315125	-1.823560	-0.000000
C	-0.119059	0.975380	0.000000
C	-0.218432	2.173883	0.000000
H	-0.309091	3.232048	0.000000

____

R = CN Conformer *ac*

G4 energy: -245.9422700000; Dipole moment = 2.3066 Debye; Rotational constants (MHz), A=10456.92 B=4302.00 C=3048.03

atom	X	Y	Z
C	-1.027434	-1.294467	0.000000
H	-2.042201	-0.924512	0.000000
H	-0.854318	-2.363018	0.000000
C	0.000000	-0.443477	0.000000
O	1.294453	-0.872063	0.000000
H	1.889984	-0.115660	0.000000
C	-0.194384	0.979166	0.000000
N	-0.288311	2.133195	0.000000

____

R = CN Conformer *ap*

G4 energy: -245.9416600000; Dipole moment = 5.0896 Debye; Rotational constants (MHz), A=10531.61 B=4287.52 C=3047.04

atom	X	Y	Z
C	-1.091630	-1.192578	-0.000000
H	-2.075853	-0.746922	-0.000000
H	-1.021055	-2.274691	-0.000000
C	0.000000	-0.422362	-0.000000
O	1.293528	-0.838240	0.000000
H	1.310627	-1.802596	-0.000000
C	-0.102762	1.011791	0.000000
N	-0.199370	2.164146	0.000000

____

R = Cl Conformer *ac*

G4 energy: -613.2124470000; Dipole moment = 0.4812 Debye; Rotational constants (MHz), A=10439.87 B=5102.83 C=3427.52

atom	X	Y	Z
C	1.241723	1.114963	-0.000027
H	0.797446	2.097641	-0.000010
H	2.319539	1.016131	-0.000106

C	0.491104	0.021733	0.000032
O	0.975763	-1.235248	0.000072
H	0.246695	-1.863659	-0.000542
Cl	-1.268632	0.106570	0.000003

R = Cl Conformer *ap*

G4 energy: -613.2107530000; Dipole moment = 2.4867 Debye; Rotational constants (MHz), A=10574.30 B=5128.31 C=3453.46

atom	X	Y	Z
C	-1.169831	1.156543	0.000000
H	-0.690073	2.123019	-0.000000
H	-2.253469	1.119070	-0.000000
C	-0.456895	0.034458	0.000000
O	-0.907649	-1.237253	-0.000000
H	-1.872974	-1.217605	-0.000000
Cl	1.284592	0.042796	0.000000

R = H Conformer *ac*

G4 energy: -153.7219000000; Dipole moment = 1.9376 Debye; Rotational constants (MHz), A=64519.71 B=10432.13 C=8980.14

atom	X	Y	Z
C	1.236264	-0.074187	0.000000
H	2.081086	0.600789	0.000000
H	1.426185	-1.140713	0.000000
C	0.000000	0.410410	0.000000
O	-1.104644	-0.389765	-0.000000
H	-1.892206	0.159312	-0.000000
H	-0.195497	1.481392	-0.000000

R = H Conformer *ap*

G4 energy: -153.7235670000; Dipole moment = 1.0285 Debye; Rotational constants (MHz), A=61087.72 B=10541.83 C=8990.37

atom	X	Y	Z
C	1.213260	-0.106572	0.000000
H	2.092359	0.523463	0.000000
H	1.366082	-1.181544	0.000000
C	0.000000	0.437951	0.000000
O	-1.193502	-0.210145	-0.000000
H	-1.031656	-1.161239	-0.000000
H	-0.158331	1.512208	0.000000

R = cyclopentadienyl Conformer *ac1*

G4 energy: -346.5236080000; Dipole moment = 2.1867 Debye; Rotational constants (MHz), A=4714.54 B=1524.67 C=1170.25

atom	X	Y	Z
C	2.174050	1.162960	-0.157157
H	1.692441	2.112235	-0.343785

H	3.255256	1.131511	-0.126316
C	1.454598	0.043093	-0.006627
O	2.114419	-1.152380	0.132360
H	1.521223	-1.776535	0.563101
C	-0.000802	-0.014092	-0.014744
C	-0.913089	1.182848	0.133181
C	-0.777317	-1.120482	-0.143482
H	-0.734652	1.717360	1.075425
C	-2.291809	0.583957	0.071596
H	-0.426745	-2.133467	-0.303389
C	-2.188922	-0.748670	-0.087954
H	-3.201774	1.164067	0.143625
H	-3.006398	-1.453744	-0.170588
H	-0.754960	1.919925	-0.665829

R = cyclopentadienyl Conformer *ap1*

G4 energy: -346.5256010000; Dipole moment = 0.7771 Debye; Rotational constants (MHz), A=4735.92 B=1537.25 C=1168.88

atom	X	Y	Z
C	2.158207	1.172797	-0.000247
H	1.676962	2.140338	-0.000464
H	3.242775	1.158263	-0.000181
C	1.436825	0.040879	-0.000048
O	2.004145	-1.205350	0.000272
H	2.961892	-1.105865	-0.000204
C	-0.014601	-0.018011	0.000103
C	-0.929333	1.184677	0.000178
C	-0.779201	-1.137319	-0.000134
H	-0.768632	1.825534	0.877682
C	-2.304825	0.572732	0.000083
H	-0.400560	-2.150101	-0.000259
C	-2.192424	-0.769101	-0.000166
H	-3.218352	1.151887	0.000061
H	-3.006555	-1.483111	-0.000414
H	-0.768570	1.825934	-0.877008

R = phenyl Conformer *ac*

G4 energy: -384.6437850000; Dipole moment = 2.2694 Debye; Rotational constants (MHz), A=3729.04 B=1202.91 C=943.60

atom	X	Y	Z
C	2.461015	-1.074218	0.433307
H	2.006688	-1.952989	0.867806
H	3.541145	-1.018971	0.389380
C	1.710359	-0.053553	0.010165
O	2.324780	1.098060	-0.412161
H	1.706193	1.591869	-0.960986
C	0.229687	-0.040693	0.009747
C	-0.506678	-1.210416	-0.223191
C	-0.468712	1.154599	0.234157
C	-1.896032	-1.187277	-0.217616
H	0.021788	-2.133861	-0.431478

C	-1.860519	1.176995	0.236323
H	0.082689	2.065728	0.443594
C	-2.579392	0.006579	0.011017
H	-2.448755	-2.102034	-0.404959
H	-2.382334	2.110133	0.421429
H	-3.664021	0.023550	0.009047

——

R = phenyl Conformer *ap*

G4 energy: -384.6452970000; Dipole moment = 0.6750 Debye; Rotational constants (MHz), A=3753.38 B=1217.86 C=933.35

atom	X	Y	Z
C	-2.455518	1.119858	0.284396
H	-2.018946	2.065949	0.568070
H	-3.538722	1.063837	0.259091
C	-1.694417	0.055577	0.002009
O	-2.218668	-1.172442	-0.296663
H	-3.175623	-1.130381	-0.195740
C	-0.215884	0.039147	-0.006124
C	0.526874	1.218759	-0.152327
C	0.473170	-1.172644	0.134531
C	1.915618	1.189684	-0.135530
H	0.012748	2.161074	-0.303259
C	1.864133	-1.199566	0.149683
H	-0.091821	-2.090268	0.237048
C	2.591443	-0.019937	0.018400
H	2.472840	2.113223	-0.253686
H	2.380246	-2.147217	0.264386
H	3.676108	-0.041949	0.027162

——

R = pirrol Conformer *ac*

G4 energy: -362.5876970000; Dipole moment = 0.9975 Debye; Rotational constants (MHz), A=4898.72 B=1642.69 C=1293.89

atom	X	Y	Z
C	2.112904	-1.091130	0.392244
H	1.649274	-1.965224	0.824253
H	3.188434	-1.056209	0.293686
C	1.380431	-0.045115	0.017220
O	1.948375	1.132475	-0.359498
H	1.351888	1.580003	-0.972328
N	-0.029021	-0.033576	-0.003713
C	-0.845313	-1.104693	-0.310602
C	-0.825107	1.054904	0.319233
C	-2.149029	-0.700013	-0.184174
H	-0.411456	-2.038299	-0.626994
C	-2.136207	0.667873	0.217185
H	-0.376108	1.979968	0.641329
H	-3.018399	-1.309912	-0.375702
H	-2.993561	1.293948	0.411093

——

R = pirrol Conformer *ap*

G4 energy: -362.5885750000; Dipole moment = 1.8304 Debye; Rotational constants (MHz), A=4931.60 B=1670.40 C=1278.97

atom	X	Y	Z
C	-2.096617	1.126267	0.287421
H	-1.642302	2.050994	0.608095
H	-3.175184	1.091587	0.203016
C	-1.357493	0.050478	0.005197
O	-1.850398	-1.178565	-0.304426
H	-2.795552	-1.179040	-0.119960
N	0.041314	0.028261	-0.021925
C	0.866522	1.121782	-0.220012
C	0.829077	-1.088880	0.206316
C	2.164591	0.700361	-0.116416
H	0.446319	2.084915	-0.453943
C	2.140641	-0.701111	0.155999
H	0.368769	-2.044181	0.386303
H	3.038551	1.321599	-0.238359
H	2.993060	-1.348563	0.292712

Enethiols

R = C_2H_3 Conformer *ac*

G4 energy: -553.9435250000; Dipole moment = 1.3430 Debye; Rotational constants (MHz), A=5383.06 B=3570.89 C=2164.23

atom	X	Y	Z
C	0.849788	1.709875	-0.011769
H	0.314470	2.652062	-0.074402
H	1.931782	1.751585	-0.001502
C	0.178502	0.550184	0.026662
C	-1.284207	0.512447	0.048211
H	-1.741554	1.492920	0.172520
C	-2.071795	-0.558891	-0.061804
H	-1.675817	-1.557056	-0.209615
H	-3.150756	-0.461408	-0.026359
S	1.118465	-0.968676	-0.045175
H	0.392714	-1.660974	0.854362

R = C_2H_3 Conformer *ap*

G4 energy: -553.9433810000; Dipole moment = 1.0541 Debye; Rotational constants (MHz), A=5282.21 B=3715.19 C=2181.12

atom	X	Y	Z
C	0.872209	1.703534	-0.000171
H	0.338019	2.647640	-0.000437
H	1.954136	1.752134	-0.000240
C	0.190851	0.549847	0.000138
C	-1.272915	0.534374	0.000191
H	-1.720498	1.525986	0.001093
C	-2.065264	-0.538911	-0.000303

H	-1.678115	-1.552373	-0.001067
H	-3.144245	-0.436188	0.000195
S	0.978653	-1.052162	0.000159
H	2.242971	-0.595669	-0.001221

R = CCH Conformer *ac*

G4 energy: -552.7102720000; Dipole moment = 1.0451 Debye; Rotational constants (MHz), A=6665.10 B=3079.26 C=2113.14

atom	X	Y	Z
C	0.274859	1.776401	0.025514
H	-0.536092	2.492205	-0.027898
H	1.287802	2.158180	0.070840
C	0.022048	0.461244	0.017949
C	-1.287319	-0.083950	-0.010809
C	-2.381193	-0.588110	-0.014724
H	-3.353467	-1.015254	-0.026881
S	1.380584	-0.706836	-0.043350
H	0.742042	-1.719265	0.569968

R = CCH Conformer *ap*

G4 energy: -552.7095800000; Dipole moment = 1.2250 Debye; Rotational constants (MHz), A=6526.59 B=3152.26 C=2125.62

atom	X	Y	Z
C	0.272630	1.775586	-0.000041
H	-0.549694	2.480216	0.000250
H	1.278894	2.176462	-0.000293
C	0.028553	0.458575	0.000034
C	-1.286378	-0.082035	0.000025
C	-2.381570	-0.581772	-0.000015
H	-3.355066	-1.006420	-0.000018
S	1.280128	-0.817509	-0.000023
H	2.344405	0.007757	0.000412

R = CN Conformer *ac*

G4 energy: -568.8123260000; Dipole moment = 3.3356 Debye; Rotational constants (MHz), A=6734.48 B=3128.14 C=2143.66

atom	X	Y	Z
C	0.080614	1.778673	0.026749
H	-0.801151	2.405147	-0.026881
H	1.048258	2.264146	0.070288
C	-0.027353	0.445362	0.020095
C	-1.297512	-0.205709	-0.012488
N	-2.312296	-0.763715	-0.018203
S	1.409063	-0.610704	-0.044549
H	0.859462	-1.661994	0.590661

R = CN Conformer *ap*

G4 energy: -568.8111760000; Dipole moment = 4.7743 Debye; Rotational constants (MHz), A=6659.63 B=3175.92 C=2150.41

atom	X	Y	Z
C	0.069187	1.771814	0.000024
H	-0.825968	2.381076	-0.000115
H	1.025349	2.280768	0.000191
C	-0.021762	0.436674	-0.000021
C	-1.297923	-0.210948	-0.000047
N	-2.321057	-0.752266	0.000025
S	1.327809	-0.723886	0.000020
H	2.306052	0.200950	-0.000299

R = Cl Conformer *ac*

G4 energy: -936.0790310000; Dipole moment = 1.0295 Debye; Rotational constants (MHz), A=7281.53 B=3285.85 C=2279.93

atom	X	Y	Z
C	-0.089944	1.758500	0.033214
H	-1.042472	2.267386	-0.041981
H	0.810255	2.356856	0.103497
C	-0.010850	0.430910	0.027952
Cl	-1.462364	-0.553487	-0.015760
S	1.530286	-0.433166	-0.059989
H	1.212606	-1.420763	0.799221

R = Cl Conformer *ap*

G4 energy: -936.0782980000; Dipole moment = 2.1609 Deby; Rotational constants (MHz), A=7045.87 B=3436.43 C=2309.86

atom	X	Y	Z
C	-0.089040	1.772341	0.000006
H	-1.049875	2.269885	-0.000377
H	0.803399	2.386302	-0.000437
C	-0.006436	0.445981	0.000394
Cl	-1.447354	-0.548743	-0.000048
S	1.443577	-0.567929	-0.000041
H	2.327114	0.449387	-0.000108

R = Cyclopentadienyl Conformer *ac1*

G4 energy: -669.3933480000; Dipole moment = 1.7940 Debye; Rotational constants (MHz), A=3736.98 B=1143.34 C=885.91

atom	X	Y	Z
C	1.480914	1.766944	-0.111814
H	0.786816	2.587148	-0.257215
H	2.534118	2.012776	-0.063546
C	1.047323	0.499920	-0.019828
C	-0.355287	0.114462	-0.022418
C	-1.493229	1.106393	0.122810

C	-0.896291	-1.125326	-0.136662
H	-1.424654	1.673907	1.060273
C	-2.722318	0.241504	0.072813
H	-0.341683	-2.043951	-0.273684
C	-2.352463	-1.043908	-0.079485
H	-3.730237	0.626949	0.147776
H	-3.011372	-1.899942	-0.152279
H	-1.493399	1.852645	-0.683429
S	2.295401	-0.787396	0.020495
H	1.702109	-1.571125	0.941689

____
R = Cyclopentadienyl Conformer *ap1*

G4 energy: -669.3929870000; Dipole moment = 1.3504 Debye; Rotational constants (MHz), A=3682.99 B=1168.90 C=894.06

atom	X	Y	Z
C	1.480671	1.771852	-0.080732
H	0.774333	2.590695	-0.150452
H	2.530814	2.034758	-0.062238
C	1.052234	0.501782	-0.026774
C	-0.352729	0.120739	-0.022507
C	-1.492951	1.113403	0.077829
C	-0.887839	-1.124708	-0.091933
H	-1.433596	1.712298	0.996259
C	-2.719045	0.242597	0.052719
H	-0.332504	-2.049865	-0.179415
C	-2.344707	-1.046621	-0.045758
H	-3.728478	0.627653	0.104315
H	-3.000038	-1.907337	-0.088138
H	-1.489475	1.830921	-0.753843
S	2.184928	-0.883365	0.069984
H	3.306289	-0.179537	-0.163284

____
R = phenyl Conformer *ac*

G4 energy: -707.5138120000; Dipole moment = 1.5441 Debye; Rotational constants (MHz), A=3042.77 B=909.05 C=737.39

atom	X	Y	Z
C	-1.820447	1.645525	0.574233
H	-1.165071	2.406629	0.982325
H	-2.883264	1.855378	0.565404
C	-1.325688	0.481351	0.137385
C	0.124825	0.187778	0.065690
C	0.645968	-1.070317	0.397165
C	1.015457	1.194433	-0.336103
C	2.016021	-1.305967	0.349721
H	-0.029234	-1.858557	0.710201
C	2.384367	0.955859	-0.386368
H	0.621731	2.160921	-0.630657
C	2.890993	-0.295164	-0.041490
H	2.400765	-2.283642	0.620809
H	3.055481	1.746216	-0.706241
H	3.958480	-0.483312	-0.085420

S	-2.486048	-0.809132	-0.295134
H	-1.771097	-1.358512	-1.295675

____

R = phenyl Conformer *ap*

G4 energy: -707.5129780000; Dipole moment = 1.0061 Debye; Rotational constants (MHz), A=2996.41 B=923.89 C=748.20

atom	X	Y	Z
C	1.826845	1.635533	0.601997
H	1.162058	2.404527	0.978029
H	2.888691	1.849527	0.621950
C	1.332751	0.473465	0.162413
C	-0.121267	0.190358	0.077752
C	-0.647881	-1.063281	0.417659
C	-1.004724	1.194784	-0.343307
C	-2.018064	-1.297468	0.356083
H	0.019295	-1.850448	0.750381
C	-2.373395	0.958875	-0.403012
H	-0.605377	2.156812	-0.644307
C	-2.886193	-0.288340	-0.052303
H	-2.407279	-2.272122	0.631349
H	-3.039899	1.747980	-0.735352
H	-3.953742	-0.474112	-0.104411
S	2.359517	-0.875892	-0.401044
H	3.535547	-0.371450	0.015381

____

R = pyrrol Conformer *ac*

G4 energy: -685.4547670000; Dipole moment = 0.6187 Debye; Rotational constants (MHz), A=3803.83 B=1230.42 C=976.19

atom	X	Y	Z
C	1.411059	1.724310	-0.413939
H	0.716901	2.482722	-0.754120
H	2.458826	1.982204	-0.345294
C	0.988339	0.496635	-0.100098
N	-0.373085	0.150153	-0.025948
C	-1.377896	1.005474	0.398381
C	-0.949454	-1.047696	-0.416923
C	-2.575810	0.353639	0.276790
H	-1.128627	1.979138	0.785101
C	-2.304320	-0.946421	-0.244489
H	-0.336442	-1.841589	-0.807928
H	-3.540136	0.753535	0.550356
H	-3.024890	-1.719049	-0.464511
H	1.541209	-1.376723	1.229673
S	2.173328	-0.801685	0.186876

____

R = pyrrol Conformer *ap*

G4 energy: -685.4533760000; Dipole moment = 1.5558 Debye; Rotational constants (MHz), A=3732.31 B=1250.76 C=989.71

atom	X	Y	Z
C	1.418976	1.718516	-0.437805
H	0.717180	2.478827	-0.756163
H	2.465648	1.984869	-0.386192
C	0.993378	0.492964	-0.124264
N	-0.370306	0.156637	-0.042845
C	-1.365760	1.004065	0.414631
C	-0.954377	-1.034597	-0.445355
C	-2.566447	0.355362	0.301293
H	-1.107756	1.969563	0.815647
C	-2.305925	-0.935143	-0.247433
H	-0.350589	-1.821693	-0.863800
H	-3.525768	0.752226	0.595903
H	-3.031238	-1.702636	-0.469546
S	2.058312	-0.875539	0.302490
H	3.172605	-0.355993	-0.242178

____

Eneselenols

R = C_2H_3 Conformer *ac*

G4 energy: -2556.9086980000 ; Dipole moment = 1.2709 Debye; Rotational constants (MHz), A=4742.04 B=2364.85 C=1590.10

atom	X	Y	Z
C	-0.505358	2.040437	0.001476
H	-1.413282	2.633912	-0.065084
H	0.434767	2.577023	0.029427
C	-0.573703	0.703881	0.033227
C	-1.850723	-0.005907	0.033697
H	-2.712452	0.655060	0.124384
C	-2.053617	-1.321388	-0.057177
H	-1.236425	-2.025061	-0.165618
H	-3.056940	-1.731369	-0.041181
Se	1.092554	-0.275730	-0.028775
H	0.737886	-1.236883	1.029079

____

R = C_2H_3 Conformer *ap*

G4 energy: -2556.9084870000; Dipole moment = 1.0948 Debye; Rotational constants (MHz), A=4609.05 B=2490.74 C=1616.94

atom	X	Y	Z
C	-0.839354	1.965720	0.000000
H	-0.444424	2.977431	0.000000
H	-1.917062	1.862040	0.000000
C	-0.000000	0.924080	0.000000
C	1.449249	1.106878	0.000000
H	1.758423	2.150956	0.000000
C	2.382610	0.153487	0.000000

H	2.138892	-0.904003	0.000000
H	3.437403	0.402206	0.000000
Se	-0.614404	-0.907133	0.000000
H	-2.038509	-0.547096	0.000000

____

R = CCH Conformer *ac*

G4 energy: -2555.6757230000; Dipole moment = 0.9236 Debye; Rotational constants (MHz), A=5896.50 B=2043.79 C=1523.13

atom	X	Y	Z
C	-0.671150	1.927935	0.029958
H	-1.629339	2.431419	-0.031197
H	0.218888	2.543469	0.084630
C	-0.605859	0.591787	0.027526
C	-1.735188	-0.258919	-0.010303
C	-2.673250	-1.015141	-0.023420
H	-3.508607	-1.670610	-0.043141
Se	1.127538	-0.273913	-0.025636
H	0.695451	-1.465200	0.718777

____

R = CCH Conformer *ap*

G4 energy: -2555.6753470000; Dipole moment = 1.1124 Debye; Rotational constants (MHz), A=5647.01 B=2127.56 C=1545.34

atom	X	Y	Z
C	-0.703668	1.952182	-0.000078
H	-1.683316	2.416417	-0.000016
H	0.159991	2.605403	-0.000332
C	-0.594994	0.619137	0.000136
C	-1.706408	-0.260615	0.000160
C	-2.615734	-1.050675	0.000012
H	-3.429315	-1.733408	-0.000629
Se	1.080935	-0.344401	-0.000022
H	1.925687	0.861050	0.000344

____

R = CN Conformer *ac*

G4 energy: -2571.7778300000; Dipole moment = 3.5792 Debye; Rotational constants (MHz), A=6029.01 B=2042.71 C=1531.29

atom	X	Y	Z
C	0.835783	1.863101	0.031171
H	1.835290	2.278293	-0.029161
H	0.004059	2.555715	0.083992
C	0.644446	0.540556	0.028683
C	1.724814	-0.385717	-0.013026
N	2.583036	-1.163893	-0.027679
Se	-1.128917	-0.216604	-0.025417
H	-0.767669	-1.429849	0.722131

____

R = CN Conformer *ap*

G4 energy: -2571.7769340000; Dipole moment = 4.6536 Debye Rotational constants (MHz), A=5847.76 B=2103.23 C=1546.88

atom	X	Y	Z
C	-0.830376	1.894735	0.000000
H	-0.437171	2.904823	0.000000
H	-1.906857	1.776150	0.000000
C	0.000000	0.847451	0.000000
C	1.416648	1.017359	0.000000
N	2.570059	1.122394	0.000000
Se	-0.506286	-1.010762	0.000000
H	-1.950306	-0.729092	0.000000

R = Cl Conformer *ac*

G4 energy: -2939.0437350000; Dipole moment = 1.1045 Debye; Rotational constants (MHz), A=6815.17 B=1971.52 C=1538.38

atom	X	Y	Z
C	-0.886953	1.786491	0.038696
H	-1.910832	2.131881	-0.040620
H	-0.100877	2.527885	0.116439
C	-0.591134	0.491043	0.037187
Cl	-1.851917	-0.721031	-0.021327
Se	1.216330	-0.142156	-0.032362
H	1.007616	-1.234130	0.931770

R = Cl Conformer *ap*

G4 energy: -2939.0433930000; Dipole moment = 1.9978 Debye; Rotational constants (MHz), A=6533.84 B=2070.30 C=1572.15

atom	X	Y	Z
C	0.954052	1.801868	-0.000079
H	1.998897	2.087022	0.000436
H	0.211349	2.590195	-0.000563
C	0.594764	0.524215	-0.000120
Cl	1.787273	-0.756084	0.000060
Se	-1.177746	-0.202682	-0.000049
H	-1.843412	1.110902	0.001986

R = H Conformer *ac*

G4 energy: -2479.5565850000; Dipole moment = 1.1554 Debye; Rotational constants (MHz), A=45677.00 B=3994.96 C=3693.57

atom	X	Y	Z
C	2.136992	-0.284645	0.037242
H	3.147347	0.101859	-0.049855
H	2.033377	-1.356373	0.172008
C	1.092261	0.532576	-0.028881
H	1.203246	1.602810	-0.161340
H	-1.283753	1.158604	0.522917

Se	-0.719875	-0.088073	-0.015703

____

R = H Conformer *ap*

G4 energy: -2479.5567730000; Dipole moment = 1.0093 Debye; Rotational constants (MHz), A=47527.38 B=3921.33 C=3622.45

atom	X	Y	Z
C	0.304899	-2.154636	-0.000000
H	-0.110515	-3.157230	-0.000000
H	1.386597	-2.073971	-0.000000
C	-0.496609	-1.095562	-0.000000
H	-1.577320	-1.199108	-0.000000
H	1.451498	0.504221	-0.000000
Se	-0.000000	0.747861	0.000000

____

R = cyclopentadienyl Conformer *ac1*

G4 energy: -2672.3588140000; Dipole moment = 1.7382 Debye; Rotational constants (MHz), A=3334.06 B=766.26 C=628.43

atom	X	Y	Z
C	0.657335	2.089496	0.091283
H	-0.146515	2.808424	0.215267
H	1.664304	2.483688	0.041273
C	0.402184	0.775353	0.016359
C	-0.927273	0.192197	0.020311
C	-2.195959	1.014833	-0.112140
C	-1.287687	-1.112488	0.124976
H	-2.297009	1.747706	0.699968
C	-3.289639	-0.016071	-0.064629
H	-0.606089	-1.943538	0.246174
C	-2.740558	-1.237383	0.074687
H	-4.342381	0.223293	-0.132395
H	-3.271297	-2.178649	0.142140
H	-2.213526	1.592596	-1.045704
Se	1.942767	-0.402627	0.000077
H	1.448006	-1.279825	-1.074414

____

R = cyclopentadienyl Conformer *ap1*

G4 energy: -2672.3583610000; Dipole moment = 1.4554 Debye; Rotational constants (MHz), A=3224.78 B=795.35 C=642.02

atom	X	Y	Z
C	0.652331	2.128976	0.095938
H	-0.170917	2.832948	0.157246
H	1.649692	2.549120	0.080440
C	0.418356	0.811037	0.048076
C	-0.906043	0.214066	0.034539
C	-2.186321	1.015240	-0.101107
C	-1.244106	-1.097790	0.124161
H	-2.306723	1.739960	0.715447
C	-3.262302	-0.035113	-0.072930

H	-0.554442	-1.924198	0.241786
C	-2.694129	-1.248404	0.057967
H	-4.318365	0.187096	-0.145540
H	-3.208301	-2.199634	0.110439
H	-2.205495	1.598765	-1.031008
Se	1.868512	-0.471314	-0.044157
H	2.918405	0.512534	0.252661

____

R = phenyl Conformer *ac*

G4 energy: -2710.479049; Dipole moment = 1.4262 Debye; Rotational constants (MHz), A=2705.49 B=616.69 C=525.80

atom	X	Y	Z
C	1.016694	2.010535	-0.625541
H	0.254482	2.696436	-0.981230
H	2.042130	2.360140	-0.640092
C	0.686456	0.781777	-0.216763
C	-0.707343	0.300295	-0.104695
C	-1.695867	1.165101	0.389855
C	-1.085051	-0.993468	-0.489187
C	-3.021729	0.755168	0.477536
H	-1.410434	2.156106	0.725062
C	-2.412416	-1.399700	-0.405127
H	-0.332008	-1.674154	-0.869633
C	-3.386314	-0.528410	0.077864
H	-3.769538	1.437237	0.868661
H	-2.686304	-2.401788	-0.718405
H	-4.419696	-0.850390	0.150802
Se	2.132160	-0.445578	0.163406
H	1.461353	-1.121724	1.285400

____

R = phenyl Conformer *ap*

G4 energy: -2710.4783680000; Dipole moment = 1.0294 Debye; Rotational constants (MHz), A=2604.36 B=638.57 C=541.39

atom	X	Y	Z
C	-1.029635	2.040868	0.656868
H	-0.252046	2.732609	0.964186
H	-2.051111	2.397685	0.705126
C	-0.709704	0.806175	0.263469
C	0.683333	0.322228	0.131071
C	1.663807	1.164998	-0.413576
C	1.062353	-0.962229	0.545552
C	2.984079	0.743207	-0.521426
H	1.375478	2.147494	-0.770229
C	2.384304	-1.382127	0.436394
H	0.319767	-1.625028	0.975909
C	3.350267	-0.531975	-0.095704
H	3.726694	1.408809	-0.949056
H	2.659191	-2.376786	0.771426
H	4.379523	-0.862835	-0.185063
Se	-2.038327	-0.508693	-0.229145
H	-3.187222	0.266729	0.262727

R = pyrrol Conformer *ac*

G4 energy: -2688.4194820000; Dipole moment = 0.6194 Debye; Rotational constants (MHz), A=3358.98 B=822.89 C=687.27

atom	X	Y	Z
C	-0.585603	2.062023	0.422582
H	0.225656	2.718895	0.714738
H	-1.582350	2.477591	0.367780
C	-0.359592	0.776586	0.143907
N	0.927547	0.224467	0.048846
C	2.034772	0.902463	-0.439778
C	1.331809	-1.031708	0.471032
C	3.124796	0.082253	-0.325553
H	1.920579	1.887769	-0.859063
C	2.679679	-1.142260	0.256332
H	0.619656	-1.710577	0.907850
H	4.128299	0.321982	-0.642148
H	3.283554	-2.006297	0.487234
Se	-1.857551	-0.412722	-0.095228
H	-1.286633	-1.124234	-1.251692

R = pyrrol Conformer *ap*

G4 energy: -2688.4184040000; Dipole moment = 1.4006 Debye; Rotational constants (MHz), A=3217.91 B=851.61 C=706.08

atom	X	Y	Z
C	-0.591225	2.095511	0.448535
H	0.236275	2.748772	0.699222
H	-1.582995	2.524835	0.418993
C	-0.377808	0.805657	0.189463
N	0.906733	0.247796	0.077600
C	1.999266	0.890681	-0.481677
C	1.313799	-0.995518	0.536620
C	3.085022	0.062871	-0.374857
H	1.877384	1.858416	-0.938121
C	2.651377	-1.130751	0.274109
H	0.616326	-1.643282	1.040130
H	4.078136	0.278411	-0.737982
H	3.255623	-1.990476	0.519474
Se	-1.778744	-0.477134	-0.163264
H	-2.833165	0.340605	0.452884
